# Acetylation of PAX7 controls muscle stem cell self-renewal and differentiation potential in mice

**DOI:** 10.1038/s41467-021-23577-z

**Published:** 2021-05-31

**Authors:** Marie-Claude Sincennes, Caroline E. Brun, Alexander Y. T. Lin, Tabitha Rosembert, David Datzkiw, John Saber, Hong Ming, Yoh-ichi Kawabe, Michael A. Rudnicki

**Affiliations:** 1 Sprott Centre for Stem Cell Research, Regenerative Medicine Program, Ottawa Hospital Research Institute, Ottawa, ON Canada; 2 Department of Cellular and Molecular Medicine, Faculty of Medicine, University of Ottawa, Ottawa, ON Canada

**Keywords:** Acetylation, Transcriptional regulatory elements, Muscle stem cells

## Abstract

Muscle stem cell function has been suggested to be regulated by Acetyl-CoA and NAD+ availability, but the mechanisms remain unclear. Here we report the identification of two acetylation sites on PAX7 that positively regulate its transcriptional activity. Lack of PAX7 acetylation reduces DNA binding, specifically to the homeobox motif. The acetyltransferase MYST1 stimulated by Acetyl-CoA, and the deacetylase SIRT2 stimulated by NAD +, are identified as direct regulators of PAX7 acetylation and asymmetric division in muscle stem cells. Abolishing PAX7 acetylation in mice using CRISPR/Cas9 mutagenesis leads to an expansion of the satellite stem cell pool, reduced numbers of asymmetric stem cell divisions, and increased numbers of oxidative IIA myofibers. Gene expression analysis confirms that lack of PAX7 acetylation preferentially affects the expression of target genes regulated by homeodomain binding motifs. Therefore, PAX7 acetylation status regulates muscle stem cell function and differentiation potential to facilitate metabolic adaptation of muscle tissue.

## Abstract (web summary)

The acetyltransferase MYST1 stimulated by acetyl-CoA, and the deacetylase SIRT2 stimulated by NAD+, regulate PAX7 acetylation in muscle stem cells, which in turn, regulates stem cell self-renewal and regeneration following injury in mouse skeletal muscle.

## Introduction

Satellite cells are responsible for the post-natal growth and regenerative capacity of skeletal muscle^1^. Satellite cell function is highly dependent on cellular metabolism. For example, satellite cell activation is associated with a shift from fatty acid oxidation to glycolysis as a main energy-producing pathway^2,3^. This shift is accompanied by the increased availability of glucose-derived acetyl-CoA, leading to elevated histone acetylation levels^4^, and by a decrease in NAD+ availability, which drops the activity of Class III HDACs, or sirtuins^2^. Although acetyl-CoA and NAD+ availability influence post-translational modification of histones during satellite cell activation, the mechanism of action on quiescent stem cells remains unclear.

All satellite cells express the nodal transcription factor PAX7, which is indispensable for satellite cell maintenance and regenerative capacity in adult muscle^5–9^. PAX7 is a master regulator of satellite cell function as it controls the expression of genes promoting satellite cell survival and proliferation, while inhibiting their differentiation^10,11^. One well-characterized PAX7 target gene is the myogenic regulatory factor MYF5, a basic helix-loop-helix (bHLH) transcription factor^10,12,13^. While the role of PAX7 as a master regulator of satellite cell identity is clear, its upstream regulation has yet to be fully explored. PAX7 methylation by CARM1 during asymmetric division has been shown to promote PAX7 interaction with MLL1/2 to activate *Myf5* transcription in the committed daughter cell^12–15^. This highlights the importance of post-translational regulation of PAX7 function.

In this study, we highlight a regulatory pathway controlling PAX7 function in satellite cells. We identify two lysine acetylation sites that modulate PAX7 transcriptional activity in satellite cells. We demonstrate that these acetylation events induce preferential binding to homeodomain motifs, affecting PAX7 gene targeting. Further, we discover that the acetyltransferase MYST1 and the deacetylase SIRT2 are responsible for the deposition and removal of these acetylation marks on PAX7 protein, respectively. Disruption of PAX7 acetylation, through mutagenesis or depletion of MYST1 or SIRT2, alters the balance between symmetric and asymmetric satellite cell divisions. As a result, loss of PAX7 acetylation leads to increased numbers of satellite stem cells and a regeneration program that favors the formation of oxidative type IIA myofibers. Notably, MYST1 activity is stimulated by acetyl-CoA and SIRT2 activity by NAD+. Therefore, our findings provide important insight into the mechanistic control of satellite stem cell self-renewal through the regulation of post-translational modifications on PAX7, and elucidate how metabolic cues influence stem cell function.

## Results

### PAX7 acetylation is required for high-level transcriptional activation

**Fig. 1:**
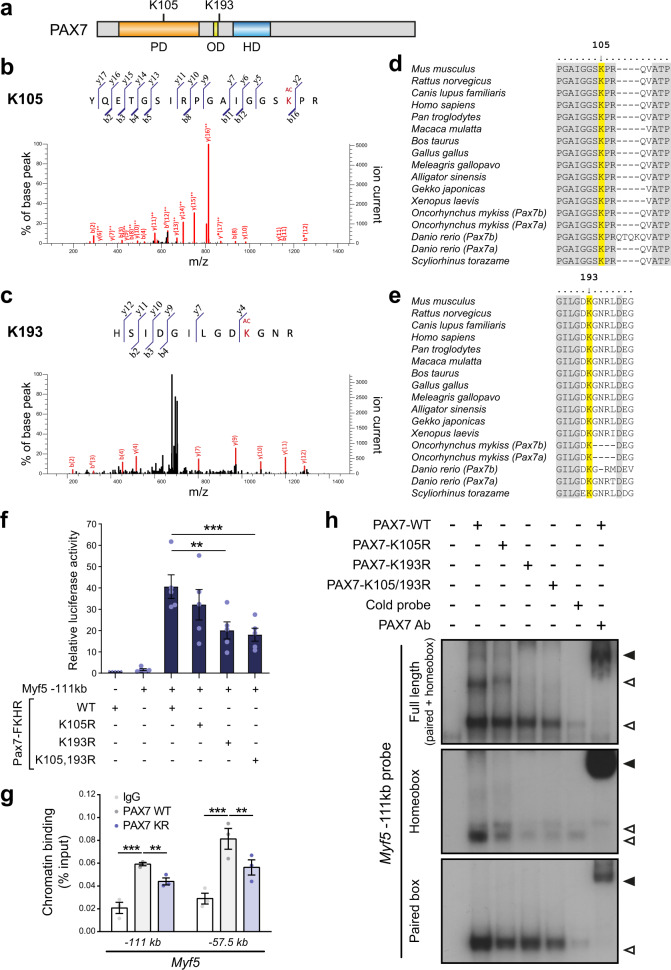
PAX7 acetylation regulates its transcriptional activity. **a** Schematic representation of PAX7 protein including the paired, octapeptide, and homeodomain, as well as the location of the two lysine residues that are acetylated. **b**, **c** Representative MS/MS spectrum of (**b**) the acetyl-lysine K105 peptide YQETGSIRPGAIGGSK(Ac)PR and (**c**) the acetyl-lysine K193 peptide HSIDGILGDK(Ac)GNR from mouse PAX7. Fragment assignments were determined using Mascot software. PAX7-FLAG was ectopically expressed in primary myoblasts, purified using anti-FLAG agarose and resolved on SDS-PAGE. The band corresponding to PAX7 protein was excised and subjected to LC-MS/MS spectrometry. **d**, **e** Amino acid sequence alignment of PAX7 K105 and K193-containing regions among the indicated species. **f** Acetylation of PAX7 controls *Myf5* expression. Cells were transfected with plasmids encoding WT or mutant PAX7-FKHR fusion as indicated, together with a luciferase reporter plasmid containing *Myf5 −111* *kb* enhancer and renilla internal control reporter plasmid DNA. Luciferase activity was normalized to control in which *Myf5* reporter plasmid was not transfected. Data are presented as mean values ± SEM (*n* = 5 independent experiments) (One-way ANOVA uncorrected Fisher’s LSD test: **p = 0.002; ***p = 0.0009). **g** PAX7 binding to chromatin is regulated by acetylation. C2C12 myoblasts were transfected with plasmids encoding FLAG-tagged WT or K105/193R PAX7 as indicated, and PAX7 recruitment at the *Myf5 −111* *kb* and *Myf5 −57.5* *kb* enhancers was determined by chromatin immunoprecipitation using anti-FLAG antibodies. Data are presented as mean values ± SEM (*n* = 3 independent experiments) (Two-way ANOVA uncorrected Fisher’s LSD test: ***p < 0.0001 *−111* *kb* IgG vs WT; **p = 0.0043 *−111* *kb* WT vs KR; ***p < 0.0001 *−57,5* *kb* IgG vs WT; **p = 0.0043 *−57,5* *kb* WT vs KR). **h** Electromobility shift assay (EMSA) showing WT and mutant PAX7 binding to full-length, homeobox motif or paired motif of the *Myf5 −111* *kb* enhancer. Binding is abrogated when a cold probe is used as a competitor. Specificity of the PAX7 binding is controlled by supershift using an anti-PAX7 antibody. Empty arrows indicate shift, while black arrows indicate supershift.

To identify post-translational modifications present on PAX7, we immunoprecipitated PAX7 from primary myoblasts and subjected the purified PAX7 protein to liquid chromatography-tandem mass spectrometry^13^ (Fig. 1a–c). This revealed that PAX7 is acetylated on two lysine residues: K105 and K193. K105, which lies within the paired domain of PAX7, is highly conserved amongst the PAX protein family in vertebrates. However, K193 is unique to PAX7, located between the paired and homeodomain, suggesting a specific role for K193 acetylation in regulating PAX7 function (Fig. 1d, e).

To determine the role of PAX7 acetylation, the two lysine residues were substituted with arginine by site-directed mutagenesis. Mutation of K105 and K193 did not affect protein stability, nuclear localization or interaction with known protein-binding partners CARM1 or ASH2L^12–14^ (Supplementary Fig. 1). We performed luciferase assays using a well-characterized PAX7 target gene, *Myf5*, as a reporter (Fig. 1f). PAX7 alone is a very poor trans-activator^16^. Therefore, we used the PAX7-FKHR fusion protein in luciferase assays. Transfection of WT-PAX7-FKHR induced a strong luciferase activity, as anticipated^10^. Mutation of lysine 105 did not significantly affect PAX7-FKHR transcriptional activity. However, both K193R and K105/193R mutations led to a 50% decrease in luciferase signal, suggesting that K193 is essential to mediate PAX7 transcriptional activity.

We then asked whether acetylation regulates PAX7 recruitment to its target genes. By chromatin immunoprecipitation in myoblasts, we observed a significant decrease in PAX7 K105/193R (PAX7-KR) recruitment to the *Myf5* enhancers *(−111* *kb* and *−57.5* *kb*) compared to PAX7-WT (Fig. 1g). Although PAX7 has the potential to be recruited to DNA via either homeobox or paired motifs, its preference for the homeobox motif has been observed both in vitro and in primary myoblasts^10^. To decipher how acetylation affects PAX7 DNA binding, we performed electrophoretic mobility-shift assays (EMSAs) using probes derived from the *Myf5 –111* *kb* enhancer, containing either a paired motif alone, a homeobox motif alone, or both (Fig. 1h). Mutation of K105 or K193 did not alter PAX7 recruitment to the paired motif. In contrast, binding to the homeobox motif was drastically reduced for PAX7-K105R, and almost completely abolished for PAX7-K193R and PAX7-K105/193R.

These experiments demonstrate that acetylation of PAX7 regulates its transcriptional activity by controlling recruitment to chromatin and more specifically, its binding to DNA via homeobox motifs.

### PAX7 acetylation levels are regulated by MYST1 and SIRT2

We next set out to identify the acetyltransferase and deacetylase controlling PAX7 activity. We focused on 9 acetyltransferases that are expressed in satellite cells and known to interact with PAX7 homologs or PAX7 protein-binding partners. All have been tested for interaction with PAX7, with particular attention paid to p300, MYST1, and TIP60, as they interact with PAX5/PAX6 and the MLL complex, respectively^17–19^ (Supplementary Fig. 2a).

**Fig. 2:**
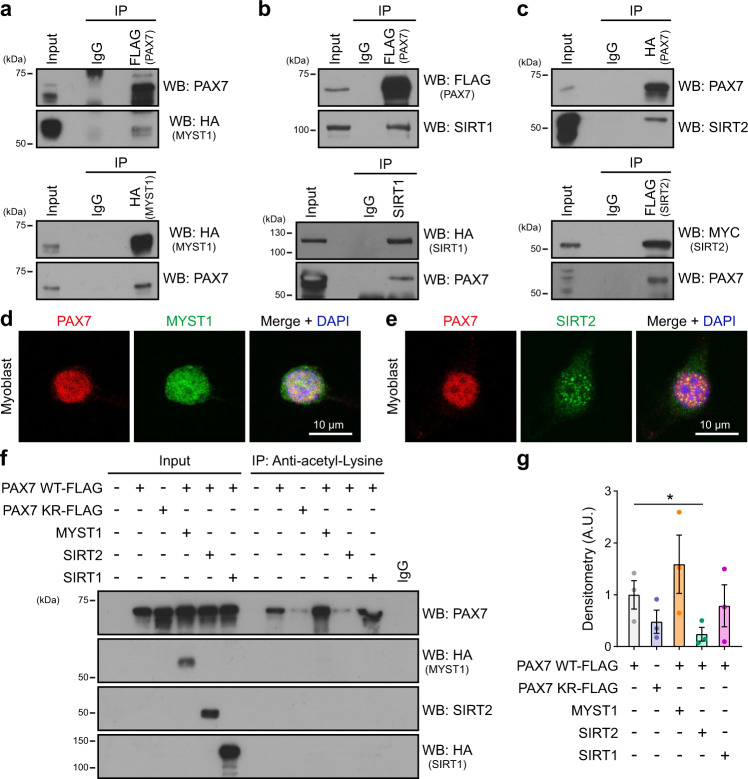
PAX7 protein interacts with MYST1, SIRT1, and SIRT2. **a** PAX7 interacts with MYST1. Cells were transfected with plasmids expressing HA-MYST1 and FLAG-PAX7, as indicated. The interaction between PAX7 and MYST1 was determined by immunoprecipitation using anti-FLAG antibodies (top) or anti-HA antibodies (bottom). Immunoblotting was performed with the indicated antibodies. **b** PAX7 interacts with SIRT1. Cells were transfected with plasmids expressing HA-SIRT1 and FLAG-PAX7, as indicated. The interaction between PAX7 and SIRT1 was determined by immunoprecipitation using anti-FLAG antibodies (top) or anti-SIRT1 antibodies (bottom). Immunoblotting was performed with the indicated antibodies. **c** PAX7 interacts with SIRT2. Cells were transfected with plasmids expressing MYC-SIRT2-FLAG and HA-PAX7, as indicated. The interaction between PAX7 and SIRT2 was determined by immunoprecipitation using anti-HA antibodies (top) or anti-FLAG antibodies (bottom). Immunoblotting was performed with the indicated antibodies. **d**, **e** Representative confocal images displaying the co-localization between PAX7 and MYST1 (**d**) or SIRT2 (**e**). Primary myoblasts were immunostained with PAX7 (red) and MYST1 or SIRT2 (green), and nuclei were counterstained with DAPI (blue). Scale bar represents 10 μm. Images are representative of ≥80% of the cells examined (*n* = 3 experiments from biologically independent samples). **f** PAX7 acetylation levels are regulated by MYST1 and SIRT2. Cells were transfected with plasmids encoding PAX7 (WT or K105/193R), MYST1, SIRT2, and SIRT1, and lysates were subjected to immunoprecipitation using anti-acetyl-lysine antibodies. Immunoblotting was performed with the indicated antibodies. **g** Quantification of the immunoblotting signals from (**f**). The densitometric analysis of the level of acetylated PAX7, relative to total PAX7 signal, is shown. Data are presented as mean values ± SEM (*n* = 3 independent experiments) (One-way ANOVA uncorrected Fisher’s LSD test: *p = 0.049).

Using co-immunoprecipitation, we detected an interaction between PAX7 and MYST1 (also known as KAT8 or MOF) (Fig. 2a). We then hypothesized that acetyltransferases and deacetylases that share the same histone target would also share non-histone targets. MYST1 specifically acetylates histone H4 lysine 16^20,21^. SIRT1 and SIRT2 are the two known deacetylases for histone H4K16^22–24^. Interestingly, we found that both SIRT1 and SIRT2 interact with PAX7 (Fig. 2b, c), suggesting they could be candidate deacetylases for PAX7. By confocal microscopy in primary myoblasts, we confirmed that PAX7 co-localized in the nucleus with both MYST1 and SIRT2, in nearly all the cells that were examined (Fig. 2d, e).

We next addressed whether MYST1, SIRT1, and SIRT2 regulate PAX7 acetylation status. Immunoprecipitation using anti-acetyl-lysine antibody revealed that PAX7 is acetylated in myoblasts, and that mutation of lysines 105 and 193 led to a decrease in PAX7 acetylation levels as anticipated (Fig. 2f, g). Expression of MYST1 led to a drastic increase in the level of PAX7 acetylation, while SIRT2 had the opposite effect. However, over-expression of SIRT1 did not change PAX7 acetylation levels, suggesting that SIRT1 does not deacetylate PAX7. Importantly, the effect of MYST1 and SIRT2 on PAX7 acetylation was almost completely abrogated when K105 and K193 were mutated (Supplementary Fig. 2b), confirming the specificity of MYST1 and SIRT2 for these residues. All together, these results support a role for MYST1 and SIRT2 as PAX7-interacting partners that regulate PAX7 acetylation levels.

### MYST1 and SIRT2 control the expression of PAX7 target genes

**Fig. 3:**
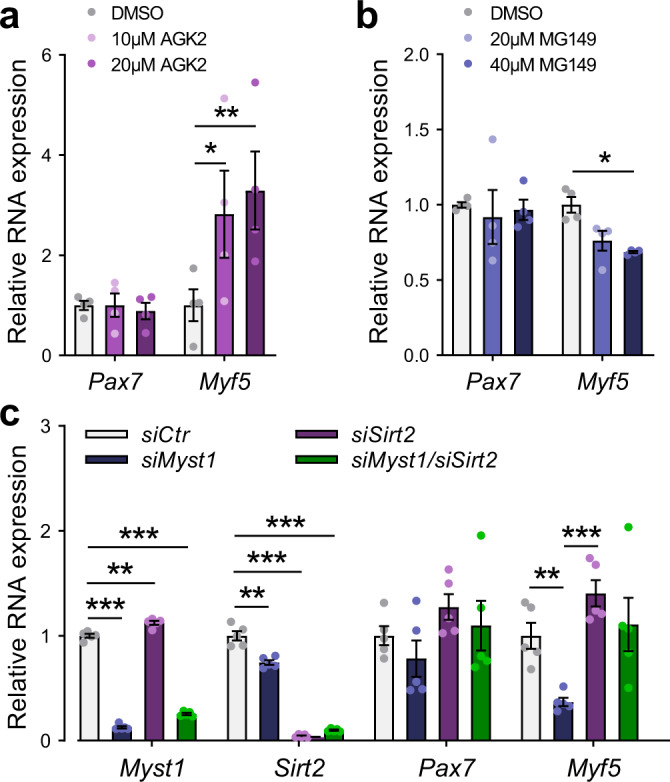
MYST1 and SIRT2 control the expression of PAX7 target genes. **a**, **b** Expression level of *Pax7* and *Myf5* in primary myoblasts after treatment for 24 h with **a** the SIRT2 inhibitor AGK2 (10 and 20 μM) or **b** the MYST1 inhibitor MG149 (20 and 40 μM), as indicated. Gene expression was determined by RT-qPCR (normalized to *Rps18* expression). Data are presented as mean values ± SEM (*n* = 6 biologically independent samples) (Two-way ANOVA uncorrected Fisher’s LSD test: *p = 0.021 in (**a**); **p = 0.0052 in (**a**) *p = 0.018 in (**b**)). **c** Expression level of *Myst1*, *Sirt2*, *Pax7*, and *Myf5* in primary myoblasts after transfection with siRNA against *Myst1* or *Sirt2*, as indicated. Gene expression was determined by RT-qPCR (normalized to *Rps18* expression). Data are presented as mean values ± SEM (*n* = 6 biologically independent samples) (Two-way ANOVA uncorrected Fisher’s LSD test: ***p < 0.0001 *siMyst1* (*Myst1*); **p = 0.0015 *siSirt2* (*Myst1*); ***p < 0.0001 *siMyst1/siSirt2* (*Myst1*); **p = 0.0023 *siMyst1* (*Sirt2*); ***p < 0.0001 *siSirt2* (*Sirt2*); ***p < 0.0001 *siMyst1/siSirt2* (*Sirt2*); **p = 0.0053 *siMyst1* (*Myf5*); ***p = 0.0006 *siSirt2* (*Myf5*)).

We then examined whether MYST1 and SIRT2 modulate the expression of the PAX7 target gene *Myf5*. First, we cultured primary myoblasts with MYST1 and SIRT2 specific inhibitors. Treatment with AGK2, a SIRT2 inhibitor, led to a drastic increase in *Myf5* expression (Fig. 3a). By contrast, we observed a significant decrease in *Myf5* expression following MG149 exposure, a MYST1 specific inhibitor (Fig. 3b). Of note, *Pax7* mRNA levels did not vary, suggesting that MYST1 and SIRT2 do not regulate the transcription of *Pax7*. Second, we transfected primary myoblasts with siRNAs against *Myst1* and *Sirt2* (Fig. 3c, Supplementary Fig. 3a, b). Knockdown of *Myst1* led to a significant decrease of *Myf5*, without affecting *Pax7* expression itself. Knockdown of *Sirt2* increased *Myf5* expression, and when primary myoblasts were co-transfected with two siRNAs targeting *Myst1* and *Sirt2*, *Myf5* expression was restored to normal levels. Thus, we conclude that MYST1 and SIRT2 regulate PAX7 target gene expression in primary myoblasts.

### MYST1 and SIRT2 regulate PAX7 acetylation in satellite cells

**Fig. 4:**
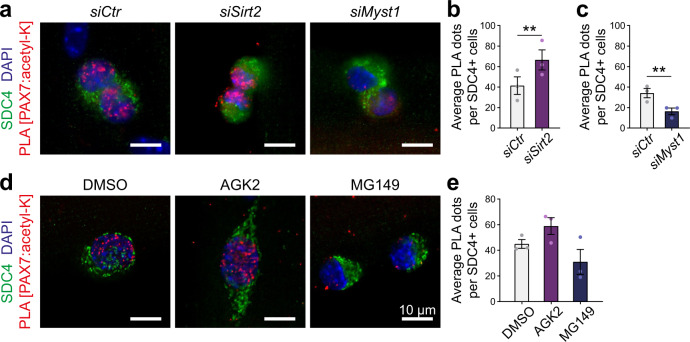
MYST1 and SIRT2 control the level of PAX7 acetylation in satellite cells. **a** Representative PAX7:acetylated-lysine PLA (red) performed on satellite cells on cultured myofibers following siRNA treatments against *Myst1* or *Sirt2*, as indicated. Satellite cells are labeled with SYNDECAN-4 (SDC4, green) and nuclei are counterstained with DAPI (blue). Scale bar represents 10 μm. Images are representative of ≥75% of the cells examined (*n* = 3 mice). **b**, **c** Quantification of the PLA signals from (**a**), represented as the mean ± SEM. The PLA was quantified by counting the number of nuclear PLA puncta for each satellite cell. **b** (*n* = 69 *siCtrl*, *n* = 61 *siMyst1* cells from 3 mice). **c** (*n* = 86 *siCtrl*, *n* = 77 *siSirt2* cells from 3 mice) (Two-tailed paired *t* test: **p = 0.0082 in (**b**); **p = 0.0096 in (**c**)). **d** Representative PAX7:acetylated-lysine PLA (red) performed on satellite cells cultured on myofibers in presence of DMSO, AGK2 or MG149, as indicated. SDC4 marks the satellite cells (green) and nuclei are counterstained with DAPI (blue). Scale bar represents 10 μm. Images are representative of ≥75% of the cells examined (*n* = 3 mice). **e** Quantification of the PLA signals from (**d**), represented as the mean ± SEM. The PLA was quantified by counting the number of nuclear PLA puncta for each satellite cell (*n* = 107 DMSO, *n* = 107 MG149, *n* = 90 AGK2 cells from 3 mice).

We next investigated whether MYST1 and SIRT2 also regulate the acetylation of PAX7 in satellite cells. To examine the acetylation status of PAX7 in satellite cells, we conducted proximity ligation assay (PLA) with antibodies recognizing PAX7 and acetyl-lysine residues. PLA specificity was confirmed in C2C12 myoblasts that were transfected with the empty vector, WT PAX7 or K105,193R PAX7 (Supplementary Fig. 3c). As an additional control, PLA was performed in isolated myofibers from mice bearing the PAX7 K193R mutation (*Pax7*^*KR*^ mice), generated using CRISPR-Cas9 gene editing. We observed a striking decrease in PLA signal at 15 h as well as at 42 h in culture, compared to *Pax7*^*WT*^ satellite cells (Supplementary Fig. 3d–f). As anticipated, isolated myofibers transfected with control siRNA presented a strong nuclear PLA signal, indicating that PAX7 is acetylated in satellite cells (Fig. 4a). By contrast, transfection with a siRNA against *Myst1* led to a 50% reduction in PLA signal, whereas transfection with a siRNA against *Sirt2* increased the PLA signal by almost 2-fold (Fig. 4b, c, Supplementary Fig. 3g). Similarly, myofibers that were exposed to MG149 (MYST1 inhibitor) presented decreased PLA signal compared to control, while AGK2-treated (SIRT2 inhibitor) fibers demonstrated the opposite effect (Fig. 4d, e, Supplementary Fig. 3h). Together, these experiments confirm that MYST1 and SIRT2 directly regulate PAX7 acetylation in satellite cells.

### MYST1 and SIRT2 regulate asymmetric satellite stem cell division

**Fig. 5:**
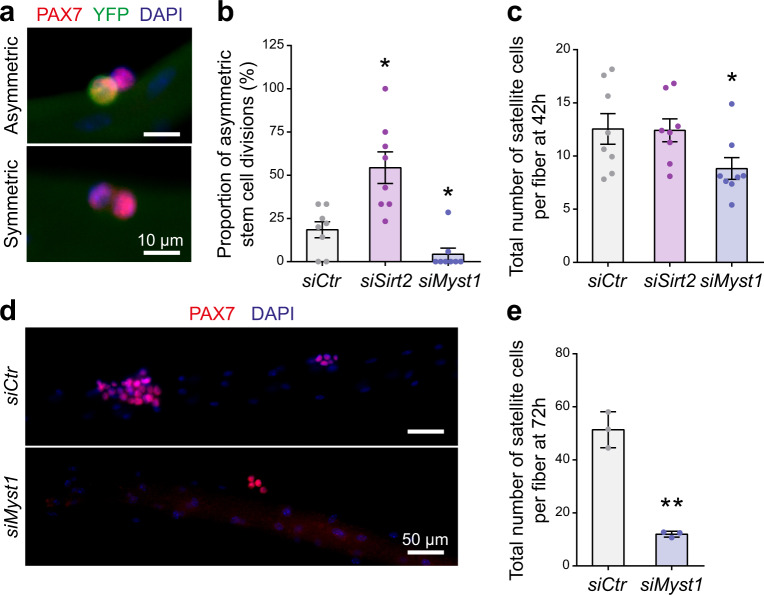
MYST1 and SIRT2 control satellite stem cell self-renewal. **a** Representative immunostaining of an asymmetric (YFP^+^/YFP^−^) and a symmetric (YFP^−^/YFP^−^) satellite stem cell division on myofibers cultured for 42 h. Myofibers were immunostained with YFP (green) and PAX7 (red), and nuclei were counterstained with DAPI (blue). Scale bar represents 10 μm. Images are representative of ≥90% of the divisions examined (*n* = 8 mice). **b** Proportion of asymmetric satellite stem cell division on myofibers cultured for 42 h. Myofibers were transfected with siRNA against *Sirt2* or *Myst1* as indicated. Data are presented as mean values ± SEM (*n* = 8 samples from independent mice) (One-way ANOVA uncorrected Fisher’s LSD test: *p = 0.0124 *siMyst1*; *p = 0.0193 *siSirt2*). **c** Number of satellite cells on myofibers cultured for 42 h and transfected as in (**b**). Data are presented as mean values ± SEM (*n* = 8 samples from independent mice) (One-way ANOVA uncorrected Fisher’s LSD test: *p = 0.0204). **d** Representative immunostaining of satellite cells on myofibers cultured for 72 h. Myofibers were transfected with siRNA against *Myst1* or control and were stained for PAX7 (red). Nuclei were counterstained with DAPI (blue). Scale bar represents 50 μm. Images are representative of ≥75% of the myofibers examined (*n* = 3 samples from independent mice). **e** Number of satellite cells on myofibers cultured for 72 h. Myofibers were transfected with siRNA against *Myst1* or control. Data are presented as mean values ± SEM (*n* = 3 samples from independent mice) (Two-tailed paired *t* test: **p = 0.007).

Given that MYST1 and SIRT2 regulate PAX7 acetylation and consequently modulate the expression of its target genes, we assessed whether MYST1 and SIRT2 govern satellite stem cell asymmetric division. To do so, we cultured isolated myofibers from *Myf5-Cre/R26R-EYFP* mice to track both the expansion and commitment of the satellite stem cells following their first division ex vivo. YFP^–^ satellite stem cells that have never expressed *Myf5* will either divide symmetrically, expanding the stem cell pool, or asymmetrically, giving rise to one self-renewing satellite stem cell (YFP^−^) and one committed satellite cell (YFP^+^)^25^ (Fig. 5a).

*Sirt2* knockdown in isolated *Myf5-Cre/R26R-EYFP* myofibers led to a significant increase in the proportion of asymmetric divisions (Fig. 5b). This shift was due to an increase in the absolute numbers of asymmetric divisions, while the numbers of symmetric divisions were unchanged (Supplementary Fig. 4). These results are consistent with our findings that SIRT2 deacetylation of PAX7 decreases transcriptional activity, *Myf5* expression, and satellite cell asymmetric divisions.

Knockdown of *Myst1*, in contrast, led to a significant decrease in the proportion of asymmetric satellite stem cell divisions (Fig. 5b), consistent with our data indicating that MYST1 positively regulates PAX7 transcriptional activity and *Myf5* expression. However, *Myst1* knockdown also decreased the total number of satellite cells at 42 h and more pronouncedly at 72 h (Fig. 5c–e), likely due to an important role of MYST1 in regulating cell cycle progression^20,21^. Nevertheless, despite the reduced number of satellite cells, the proportion of asymmetric satellite stem cell divisions was reduced by more than 5-fold in satellite cells treated with *siMyst1* (Fig. 5b). Together, our results suggest that MYST1 and SIRT2 together control the level of PAX7 acetylation, which is required for satellite cell self-renewal.

### PAX7 acetylation controls satellite cell self-renewal and regenerative potential

We assessed whether the lack of acetylation affects satellite cell self-renewal in vivo, by analyzing the phenotype of *Pax7*^*KR*^ mice. *Pax7*^*KR*^ mice were born at the expected Mendelian ratio, exhibited normal growth, and displayed no gross abnormalities. However, we observed a significant decrease in the size of the muscle fibers from *Pax7*^*KR*^ mice, suggesting a possible change in muscle fiber type composition (Supplementary Fig. 5a–e).

**Fig. 6:**
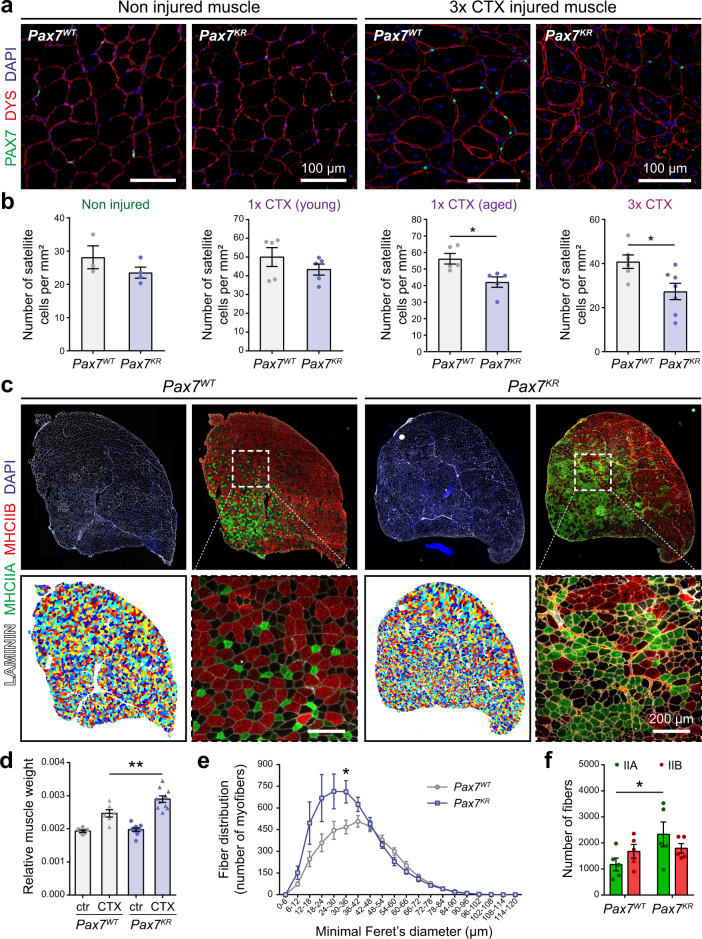
PAX7 acetylation controls satellite cell self-renewal and regenerative potential. **a** Representative immunostaining of TA muscle cross-section in uninjured muscles and after 3 rounds of CTX injury. Tissues were immunostained with DYSTROPHIN (DYS, red) and PAX7 (green), and nuclei were counterstained with DAPI (blue). Scale bar represents 100 μm. Images are representative of ≥80% of the tissues examined (*n* = 3 for *Pax7*^*WT*^ mice, *n* = 4 for *Pax7*^*KR*^ mice). **b** Quantification of the number of satellite cells in uninjured muscles and injured muscles following one CTX injection in adult and aged mice (geriatric, >21 months), and triple CTX injection in adult mice. Data are presented as mean values ± SEM (*n* = 3 for *Pax7*^*WT*^ mice, *n* = 4 for *Pax7*^*KR*^ mice) (Two-tailed unpaired *t* test: *p = 0.0139 in 1× CTX aged; *p = 0.0188 in 3× CTX). **c** Representative immunostaining of *tibialis anterior* (TA) muscle cross-section after 3 rounds of CTX injury. Top: tissues were immunostained with LAMININ (white), MHCIIA (green) and MHCIIB (red), and nuclei were counterstained with DAPI (blue). Bottom: Representative Matlab SMASH analysis of individual muscle fibers based on laminin staining, and enlarged images of cropped area of MHCIIA (green) and MHCIIB (red) immunostaining. Scale bar represents 200 μm. Images are representative of ≥80% of the tissues examined (*n* = 6 mice per group). **d** Injured TA muscle weight relative to body weight after 3 rounds of CTX injury. Data are presented as mean values ± SEM (*n* = 6 for *Pax7*^*WT*^ mice, *n* = 10 for *Pax7*^*KR*^ mice) (One-way ANOVA uncorrected Fisher’s LSD test: **p = 0.0011). **e** Minimal fiber Feret’s diameter from *Pax7*^*WT*^ and *Pax7*^*KR*^ mice after 3 consecutive rounds of CTX injury represented as absolute number of muscle fibers. Data are presented as mean values ± SEM (*n* = 6 mice per group) (Multiple unpaired *t* tests: *p = 0.0174). **f** Proportion of type IIa and type IIb fibers in *Pax7*^*WT*^ and *Pax7*^*KR*^ TA muscles after three consecutive rounds of CTX injury. Data are presented as mean values ± SEM (*n* = 5 mice per group) (Two-way ANOVA uncorrected Fisher’s LSD test: *p = 0.018).

To further assess the role of PAX7 acetylation in satellite cell function and self-renewal, we challenged the mice with cardiotoxin (CTX) injury. Satellite cell self-renewal potential decreases with aging^26^, partly due to changes in the rate of symmetric *versus* asymmetric stem cell divisions^27,28^. In addition, growing evidence suggests that satellite cells represent a phenotypically and functionally heterogenous pool, in which some rare sub-populations of cells possess a greater self-renewal capability^25,29,30^. It has recently been shown that stresses such as repeated muscle injuries, which elicit a reduction in satellite cell clonal diversity, mobilize the stem cells thought to sit at the top of the hierarchy^30,31^. In young mice, the number of satellite cells was slightly decreased in non-injured muscle from the *Pax7*^*KR*^ mouse model compared to *Pax7*^*WT*^ controls. The same was observed following one round of muscle regeneration. By contrast, one round of muscle regeneration in aged mice, or repetitive cardiotoxin (CTX)-induced muscle injuries significantly aggravated this decrease (Fig. 6a, b). Therefore, our results support a model in which loss of a single PAX7 acetylation site is sufficient to elicit a change in satellite cell numbers following repeated acute muscle regeneration cycles.

We did not observe any defect in proliferation or differentiation of *Pax7*^*KR*^ satellite cell-derived myoblasts in vitro (Supplementary Fig. 6). Consistently, we found no difference in EdU incorporation between *Pax7*^*WT*^ and *Pax7*^*KR*^ activated satellite cells in vivo (Supplementary Fig. 6). Together, these results indicate that the decreased number of satellite cells observed after multiple injuries is due to changes in self-renewal rather than proliferation defects.

We observed a significant increase in the muscle mass of triple-injured *Pax7*^*KR*^ mice, along with an increase in the numbers of small-diameter regenerated myofibers in the mutant mice (Fig. 6c–e, Supplementary Fig. 5f–i). The *Tibialis anterior* (TA) is a fast-twitch muscle, mainly composed of large glycolytic IIB fibers, as well as some smaller oxidative-glycolytic type IIA fibers^32^. Consistent with the elevated number of small myofibers, *Pax7*^*KR*^ TA regenerated muscle exhibited a significant increase in the numbers of type IIA myofibers (Fig. 6c, f). Our results suggest that a defect in PAX7 acetylation leads to changes in the differentiation potential of muscle stem cells that ultimately affect muscle fiber type composition.

### PAX7 acetylation regulates asymmetric division

**Fig. 7:**
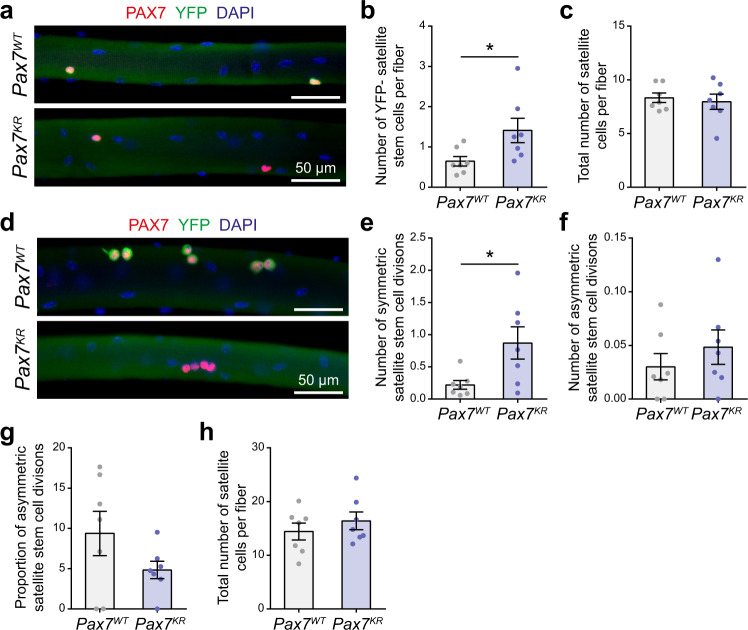
PAX7 acetylation regulates asymmetric stem cell division. **a** Representative immunostaining of satellite cells on myofibers from *Pax7*^*WT*^ and *Pax7*^*KR*^ mice, immediately after isolation (0 h). Myofibers were immunostained with YFP (green) and PAX7 (red), and nuclei were counterstained with DAPI (blue). Scale bar represents 50 μm. Images are representative of ≥75% of the myofibers examined (*n* = 7 mice per genotype). **b** Absolute numbers of YFP^−^ satellite cells from *Pax7*^*WT*^*:Myf5-Cre:R26R-EYFP* and *Pax7*^*KR*^*:Myf5-Cre:R26R-EYFP* mice immediately after isolation. Data are presented as mean values ± SEM (*n* = 7 mice per genotype) (Two-tailed unpaired *t* test: *p = 0.0368). **c** Total number of satellite cells on myofibers from *Pax7*^*WT*^*:Myf5-Cre:R26R-EYFP* and *Pax7*^*KR*^*:Myf5-Cre:R26R-EYFP* mice immediately after isolation. Data are presented as mean values ± SEM (*n* = 7 mice per genotype). **d** Representative immunostaining of satellite cells on myofibers from *Pax7*^*WT*^ and *Pax7*^*KR*^ mice, after 42 h of culture. Myofibers were immunostained with YFP (green) and PAX7 (red), and nuclei were counterstained with DAPI (blue). Scale bar represents 50 μm. Images are representative of ≥75% of the myofibers examined (*n* = 7 mice per genotype). **e**, **f** Absolute numbers per fiber of (**e**) symmetric (YFP−/YFP−) and (**f**) asymmetric (YFP+/YFP−) satellite cell doublets from *Pax7*^*WT*^*:Myf5-Cre:R26R-EYFP* and *Pax7*^*KR*^*:Myf5-Cre:R26R-EYFP* mice at 42 h post-isolation. Data are presented as mean values ± SEM (*n* = 7 mice per genotype) (Two-tailed unpaired *t* test: *p = 0.0276). **g** Proportion of asymmetric stem cell divisions after 42 h of culture. Data are presented as mean values ± SEM (*n* = 7 mice per genotype). **h** Total satellite cell numbers after 42 h of culture. Data are presented as mean values ± SEM (*n* = 7 mice per genotype).

Our results indicate that a defect in PAX7 acetylation leads to self-renewal impairment in vivo. Following cardiotoxin injury, muscle regeneration occurs through the coordinated activity of a plethora of cell types, including satellite cells, macrophages, fibro-adipogenic progenitors, and endothelial cells^1^. To pinpoint the intrinsic role of PAX7 acetylation specifically in the self-renewal process, we crossed *Pax7*^*KR*^ with *Myf5-Cre/R26R-EYFP* mice to directly measure symmetric and asymmetric satellite stem cell divisions from isolated myofibers. First, we observed a significant increase in YFP^-^ satellite stem cells directly after isolation (Fig. 7a, b), prior to myofiber culture. This indicates that a defect in PAX7 acetylation increases the number of cells that have never expressed *Myf5*. This observation suggests that in homeostatic conditions, in the absence of muscle injury, *Pax7*^*KR*^ mice underwent different cell fate decision programs during satellite stem cell division. This increase in the proportion of YFP^-^ satellite cells was maintained at 42 h. Importantly, the total number of satellite cells was the same between *Pax7*^*WT*^ and *Pax7*^*KR*^ at both time points (Fig. 7c, h), confirming that PAX7 acetylation does not affect cell proliferation, but instead specifically changes the self-renewal capacities of satellite stem cells.

We then quantified the symmetric and asymmetric satellite stem cell doublets at 42 h. If *Pax7*^*WT*^ and *Pax7*^*KR*^ mice had similar self-renewal kinetics, the increase in YFP^-^ cells at the time of isolation would lead to an equivalent increase in both symmetric and asymmetric stem cell division. While we observed an increase in the absolute number of symmetric stem cell divisions in *Pax7*^*KR*^ mice (Fig. 7d, e), we did not observe a similar increase in the number of asymmetric satellite cell doublets (YFP^-^/YFP^+^) (Fig. 7f). Therefore, the proportion of symmetric and asymmetric stem cell division is shifted in *Pax7*^*KR*^ mice (Fig. 7g). Our results indicate that in cultured myofibers, loss of PAX7 acetylation leads to alternative cell fate decisions that favor self-renewal, at the expense of commitment, most likely due to changes in regulation of PAX7 target genes in vivo.

A decrease in *Myst1* and *Sirt2* modulates the ratio of symmetric versus asymmetric satellite stem cell divisions. If this phenotype is directly mediated by PAX7 acetylation, the effect should be abrogated in *Pax7*^*KR*^ mice. Accordingly, a decrease in *Sirt2* did not affect satellite cell self-renewal in *Pax7*^*KR*^*/Myf5-Cre/R26R-EYFP* mice (Supplementary Fig. 7). Knockdown of *Myst1* reduced the number of asymmetric stem cell divisions in *Pax7*^*KR*^ fibers (Supplementary Fig. 7), although the effect was very modest compared to that observed in *Pax7*^*WT*^ fibers (Fig. 5b, Supplementary Fig. 4). The total number of satellite cells was significantly reduced after *Myst1* knockdown, both in *Pax7*^*WT*^ and in *Pax7*^*KR*^ fibers (Fig. 5c, Supplementary Fig. 7b). From this, we conclude that the effect of MYST1 on cell cycle is independent of PAX7, whereas the effect on satellite cell asymmetric division is mediated via PAX7 acetylation. Therefore, MYST1 and SIRT2 regulate satellite cell self-renewal by controlling PAX7 acetylation.

### Acetylation regulates the expression of PAX7 target genes with homeodomain binding sites

EMSA analysis indicated that PAX7 mutants lacking acetylation sites display a marked impairment in binding to homeodomain motifs (Fig. 1h). Moreover, *Pax7*^*KR*^ mice exhibit significant expansion of satellite stem cells (Fig. 7). Therefore, we examined whether PAX7 acetylation regulates the expression of target genes in satellite cells in vivo.

We first analyzed gene expression using the Biomark system with a panel of 96 different Taqman probesets, of which 80 were PAX7 target genes previously identified by ChIP-seq^10^. We compared gene expression between *Pax7*^*WT*^ and *Pax7*^*KR*^ in three different cell types: primary myoblasts, freshly isolated satellite cells from uninjured muscles (FISCs) and activated satellite cells isolated at 3 days post-injury (ASCs). In myoblasts and ASCs, there were minimal and low-order variations in PAX7 target gene expression (Supplementary Fig. 8a). In FISCs, however, we observed a global down-regulation of PAX7 target gene expression, confirming that acetylation positively regulates PAX7 transcriptional activity (Supplementary Fig. 8a).

**Fig. 8:**
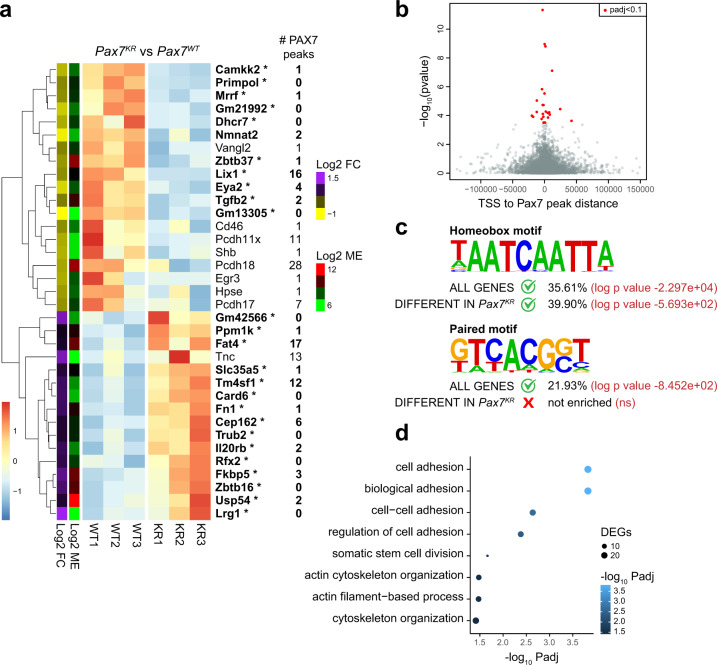
PAX7 acetylation regulates target gene expression in satellite cells. **a** Heatmap from normalized expression matrix of differentially expressed genes between *Pax7*^*WT*^ and *Pax7*^*KR*^ satellite cells. The number of PAX7 ChIP-seq peaks associated with each gene is indicated. Genes in bold with an asterisk are significantly different (Wald test followed by multiple testing correction using the Benjamini–Hochberg method to control the false discovery rate, with application of a cut-off of 0.1 (approximating to a *p*-value of 3.5 × 10^−4^) to corrected values). Heatmap coloring indicates row Z-score; color bars to the left indicate log2 fold change, and log2 mean expression. **b** Plot illustrating the distance from the closest PAX7 ChIP-seq peak and transcriptional start site (TSS) to the closest PAX7 ChIP-seq peak vs the negative log *p*-value for all PAX7 target genes. Differentially and significantly expressed genes between *Pax7*^*WT*^ and *Pax7*^*KR*^ satellite cells are highlighted in red (Wald test followed by multiple testing correction using the Benjamini–Hochberg method to control the false discovery rate, with application of a cut-off of 0.1 (approximating to a *p*-value of 3.5 × 10^−4^) to corrected values). **c** Motifs identified de novo in Pax7 peaks are similar to motifs described in Soleimani et al.^10^. The homeobox motif is enriched in PAX7 target genes (all genes), as well as in the subset that is differentially expressed in *Pax7*^*KR*^ satellite cells. In contrast, the paired motif is found only when analyzing peaks associated with all PAX7 target genes, but not when considering only the subset of genes differentially expressed in *Pax7*^*KR*^ satellite cells. Numbers indicate the percentage of peaks containing the different motifs, as well as the *p* value calculated by HOMER. **d** Gene ontology (GO) term enrichment analysis (biological process) for PAX7 target genes that are differentially expressed between *Pax7*^*WT*^ and *Pax7*^*KR*^ satellite cells.

We then undertook an unbiased approach using RNA-sequencing to compare global gene expression in freshly isolated satellite cells from *Pax7*^*WT*^ and *Pax7*^*KR*^ mice. Our data indicated that in satellite cells, removal of PAX7 acetylation leads to a molecular signature in which many genes that are downregulated are associated with one or more PAX7 ChIP-seq peaks^10^ (Fig. 8a). We validated the differential expression of a subset of these genes by RT-qPCR (Supplementary Fig. 8b). We observed that within the genes that are annotated with a PAX7 ChIP-seq peak, those that are significantly different between *Pax7*^*WT*^ and *Pax7*^*KR*^ satellite cells have the peak closer to their transcriptional start site (TSS) (most within 25 kb, all within 50 kb) (Fig. 8b). As anticipated, these differentially expressed genes presented an enrichment for binding domains containing a homeobox motif (Fig. 8c). However, while a paired motif was significantly enriched in the full list of PAX7 target genes, the HOMER motif finding analysis software failed to detect an enrichment for any paired-like motif in the list of *Pax7*^*KR*^ differentially expressed genes (Fig. 8c). These data are concordant with our EMSA experiments demonstrating that the KR mutation specifically affects PAX7 binding to the homeobox motif, and not to the paired motif (Fig. 1h). We conclude that in vivo, the acetylation of PAX7 specifically regulates the expression of genes that possess a homeobox.

To determine which molecular function is perturbed in *Pax7*^*KR*^ satellite cells, we performed Gene ontology (GO) analysis on PAX7 target genes that were affected by the KR mutation. We discovered an enrichment for genes involved in cell adhesion processes (Fig. 8d), suggesting that *Pax7*^*KR*^ satellite cells have intrinsic perturbations in their adhesion properties. Interestingly, while most genes involved in cell adhesion were downregulated in *Pax7*^*KR*^ satellite cells, we identified some genes that were significantly up-regulated, suggesting that the adhesion properties are modified, rather than lost, in the mutant cells. Gene set enrichment analysis (GSEA) generated similar results, with higher expression of genes related to the apical surface in *Pax7*^*WT*^ satellite cells, and higher expression of genes associated with epithelial-mesenchymal transition in *Pax7*^*KR*^ satellite cells (Supplementary Fig. 8c), suggesting that the KR mutation changes satellite cell properties associated with cell polarity and adhesion. Therefore, we conclude that a defect in PAX7 acetylation leads to changes in the expression of genes that regulate different molecular processes such as cell polarity and cell adhesion, consequently modulating the self-renewal properties of satellite stem cells.

Recent evidence indicates that the stem cell isolation process leads to gene expression changes reflecting partial satellite cell activation^33–36^. We asked whether the changes observed in freshly isolated satellite cells would be mirrored in cells isolated using a technique that better preserves stem cell quiescence^33^ (Supplementary Fig. 9a, b). We found that some differences in gene expression were conserved in quiescent satellite cells, but not all (Supplementary Fig. 9c). This suggests that the acetylation of PAX7 most likely regulates gene expression in a dynamic manner, with a subset of genes being regulated in the quiescent state, and some other genes being regulated at the exit of quiescence, when satellite cells prime the self-renewal process.

Together, these data suggest that PAX7 target genes containing a homeobox motif are positively regulated by PAX7 acetylation. We also conclude that PAX7 acetylation markedly affects target gene expression in freshly isolated satellite cells, and to a smaller extent in proliferating cells (ASCs and myoblasts). Taken together, our data indicate that acetylation is required for PAX7 activity in satellite cells, as acetylation influences PAX7 target gene expression, satellite cell self-renewal and regenerative capacity.

## Discussion

We have found that the acetylation of PAX7 protein regulates its transcriptional activity and controls PAX7 target gene expression. Our analysis of the *Pax7*^*KR*^ mouse model revealed that mutation of lysine 193 is sufficient to imbalance the ratio of symmetric *versus* asymmetric satellite stem cell divisions, which is specifically mediated by MYST1 and SIRT2. A shift in the proportion of symmetric vs asymmetric satellite cell divisions markedly affects muscle regeneration^13,14,27,37,38^. Here we show that a defect in PAX7 acetylation triggers an expansion of the satellite stem cell pool and shifts their differentiation potential. This disruption of self-renewal ultimately leads to a decrease in satellite cell numbers following cardiotoxin injury.

Although many studies have reported on the metabolic switch that accompanies the satellite cell activation process^2,3^, the metabolic pathways controlling satellite cell self-renewal are not well described. Quiescent satellite cells are metabolically heterogeneous, with the satellite stem cell population being more dormant and less metabolically active^29,39^. This suggests that satellite stem cells and committed satellite cells have distinct metabolic requirements. Here we show that acetylation, regulated by MYST1 and SIRT2, is a key determinant in regulating satellite cell self-renewal, suggesting that acetyl-CoA and NAD+ are likewise important drivers of this process.

Our results demonstrate that satellite cell self-renewal is controlled by the acetyltransferase MYST1 and the deacetylase SIRT2. We have demonstrated that inhibition of MYST1 and SIRT2, by drug or by siRNA treatment, directly modulates PAX7 acetylation levels. Treatment with drugs that block MYST1 and SIRT2 activity, such as MG149 and AGK2, acts on the expression of *Myf5*, a PAX7 target gene. Whether this effect is abolished in *Pax7*^*KR*^ myoblasts remains to be determined. In contrast, we have demonstrated that in *Pax7*^*KR*^ isolated myofibers, treatment with siRNA against *Myst1* and *Sirt2* has no effect on symmetric and asymmetric stem cell divisions, providing strong evidence of a causal relationship between the enzymes (MYST1 and SIRT2), the acetylation of PAX7 and the effects on satellite cell function.

MYST1 and SIRT2 share the same histone target for acetylation/de-acetylation. Indeed, MYST1 is the primary acetyltransferase mediating histone H4 lysine 16 acetylation^20,21^, associated with chromatin structure regulation, euchromatin maintenance, and active gene transcription^40–42^. This mark is removed specifically by SIRT1 and SIRT2 during the G2/M transition^22–24^. The antagonism between MYST1 and sirtuins is also observed for non-histone targets, including p53^43–45^ and MYST1 itself^46^. Our work adds PAX7 to the list of proteins that are co-regulated by MYST1 and sirtuins.

Interestingly, SIRT2 regulates self-renewal and commitment in other stem cell types. For example, knockdown of *Sirt2* leads to increased glycolysis in primed human pluripotent stem cells^47^. Interestingly, a shift towards glycolysis is a feature of satellite cell activation and commitment^2,3^. Accordingly, our results show that knockdown of *Sirt2* leads to an increase in asymmetric cell divisions, giving rise to committed satellite cells. Sirtuin activity depends on NAD +, and loss of nicotinamide phosphoribosyltransferase (NAMPT), the rate limiting enzyme in NAD+ synthesis, leads to a decrease in neural stem cell self-renewal^48^. *Nampt* over-expression is also linked to increased stemness characteristics in cancer cells^49,50^. Therefore, in keeping with our findings, SIRT2 activity is linked to stem cell self-renewal.

Little is known about the role of *Myst1* in adult stem cell biology. *Myst1*-null embryos do not progress through the blastocyst stage, most likely due to a complete absence of H4K16 acetylation^51,52^. *Myst1*-null ES cells exhibit extensive chromatin compaction leading to cell cycle arrest and cell death^53^. At the adult stage, *Myst1* deletion in hematopoietic cells results in a drastic loss of hematopoietic stem cells and in hematopoietic failure^54^. One can speculate that *Myst1* function is crucial in most stem cell types including muscle stem cells.

PAX3 and PAX7 display different chromatin-binding affinity in primary myoblasts^10^. PAX7 shows a preference for homeobox binding relatively to PAX3, and our data indicate that acetylation of lysine 193 is sufficient to alter PAX7 DNA binding to the homeobox and to regulate the expression of homeobox-containing target genes in vivo, specifically in freshly isolated satellite cells and not in proliferating cells (ASCs or myoblasts). Therefore, acetylation appears to be one regulatory mechanism that confers chromatin-binding specificity to PAX7, leading to changes in transcriptional networks that drive satellite cell self-renewal. How PAX7 acetylation can also affect its function as a pioneer factor remains to be determined^55,56^.

RNA-sequencing analyses indicate that in vivo, a defect in PAX7 acetylation leads to an impairment in the expression of PAX7 target genes, specifically in the subset that harbors a homeobox motif. These genes share a role in cellular adhesion. The interaction and cross-talk between satellite cells and the extra-cellular matrix (ECM) is critical to maintain the ability of the satellite cell to self-renew, proliferate, and differentiate^1^. In addition, the satellite cell itself expresses different matrix proteins to maintain the ECM structure. Our laboratory previously determined that proteins controlling satellite cell adhesion to its niche, such as fibronectin and dystrophin, regulate satellite cell self-renewal by shifting the ratio of symmetric and asymmetric satellite stem cell divisions^37,57^. This suggests the exciting possibility that the acetylation of PAX7 regulates satellite cell interaction with its niche, leading to changes in satellite cell self-renewal and cell fate decisions.

This study provides important insight into how a single post-translational modification on the PAX7 protein positively regulates transcription and significantly impacts satellite cell function. We also identified two upstream regulators of PAX7, MYST1, and SIRT2, which control satellite cell self-renewal and commitment. This work identifies PAX7 acetylation status as a key effector of satellite cell self-renewal and opens perspectives on how metabolic cues mediate satellite cell function.

## Methods

### Animal models

Housing, husbandry, and all experimental protocols for mice used in this study were performed in accordance with the guidelines established by the University of Ottawa Animal Care Committee, which is based on the guidelines of the Canadian Council on Animal Care. Protocols were approved by the Animal Research Ethics Board of the University of Ottawa. Six to eight-week-old mice were used for all the experiments, except for those performed in aged mice (more than 21 months). The *Myf5-Cre:R26R-EYFP* mice were F1 progeny from *Myf5-Cre* and *R26R-EYF* crossing^25^. *Pax7*^*KR*^ mice were generated by direct delivery of Cas9 reagents to *C57BL/6J* mouse (The Jackson Laboratory, #000664) zygotes essentially as described in ref. ^58^ at The Centre for Phenogenomics (Toronto, ON, Canada). Briefly, the gRNA was designed by identifying the PAM nearest to the desired mutation site. Specificity of the gRNA was evaluated using the online tool available at crispr.mit.edu. The gRNA with the desired spacer sequence (Supplementary Table 1) was synthesized by in vitro transcription from a PCR-derived template. A mix of 20 ng/µL Cas9 mRNA (ThermoFisher A29378), 10 ng/µL gRNA, and 10 ng/µL single-strand oligonucleotide template (Supplementary Table 1, mutated nucleotides indicated in lowercase) was microinjected into *C57BL/6J* zygotes. Injected zygotes were incubated in KSOM AA media (Zenith Biotech, ZEKS-50) at 37 °C with 6% CO_2_ until same-day transfer into CD-1 (Charles River Labs, Strain 022) surrogate host mothers. Tail tissue biopsies from born pups were taken for DNA isolation and genotyping (Supplementary Table 1). Founders were crossed with WT *C57BL/6J* mice. F1 heterozygous mice were interbred to generate F2 homozygous mice and WT littermates used as controls.

### Cell Lines

C2C12 cells were used for co-immunoprecipitation and ChIP experiments, COS-7 cells were used for luciferase assays, and Sf9 cells were used to produce PAX7 protein for EMSAs. C2C12, COS-7, and Sf9 cells were purchased from and authenticated by ATCC. These cells were not contaminated by mycoplasma as determined by the MycoSensor PCR Assay Kit (Agilent Technologies). C2C12 and COS-7 cells were cultured at 37 °C in Dulbecco’s Modified Eagle’s Medium (DMEM) supplemented with 10% fetal bovine serum, and 1% penicillin/streptomycin, while Sf9 cells were cultured in SF-900 III SFM media (Life Technologies).

### Primary cell cultures

*Extensor digitorum longus* (EDL) myofibers were isolated from *Myf5-Cre:R26R-EYFP* and from *Pax7*^*KR*^*:Myf5-Cre:R26R-EYFP* mice. EDL myofibers were cultured at 37 °C in DMEM (Gibco) supplemented with 20% fetal bovine serum, 1% chick embryo extract, and 2.5 ng/ml bFGF^59^. Primary myoblasts were purified by magnetic cell separation (MACS)^60^. Cells were cultured at 37 °C in Ham’s F-10 medium (Wisent) supplemented with 20% fetal bovine serum, 1% penicillin/streptomycin, and 2.5 ng/ml bFGF. Differentiation of primary myoblasts was induced by incubating the cells in 50% Ham’s F-10 medium (Wisent), 50% DMEM (Gibco), 5% horse serum and 1% penicillin/streptomycin.

### Luciferase assays

COS-7 cells were transfected with pcDNA3-PAX7-FKHR (WT or K105, 193R), pGL4-Myf5-111kb (expressing firefly luciferase under the control of *Myf5* enhancer) and pRL (expressing renilla luciferase in all transfected cells) using Lipofectamine 2000 (Life Technologies). 48 h post-transfection, cells were lysed and luciferase activity was measured with the Dual-Luciferase Reporter Assay System (Promega) according to the manufacturer’s protocol. Firefly luciferase activity was normalized to renilla luciferase internal control. Results are presented as fold increase in luciferase activity compared to control cells that do not express the *Myf5* reporter.

### Chromatin immunoprecipitation (ChIP)

Transfected C2C12 cells were cross-linked using 1% formaldehyde in PBS for 10 min. Glycine was added to a final concentration of 0.125 M for 5 min, followed by centrifugation. The cell pellet was washed in PBS and subsequently resuspended in ChIP lysis buffer (50 mM Tris-HCl pH 8.0, 10 mM EDTA, 0.5% SDS). Chromatin was fragmented using a Covaris sonicator. Before ChIP, equal volumes of ChIP dilution buffer (20 mM Tris-HCl pH 8.0, 1.2 mM EDTA, 1.1% Triton X-100, 200 mM NaCl) was added. Immunoprecipitation was performed using 0.5 mg of chromatin and anti-Flag agarose beads (Sigma) for 2 h at 4 °C. Antibody/protein/DNA complexes were collected, washed and eluted, and cross-links were reversed according to the manufacturer’s instructions. DNA was purified by phenol/chloroform purification using linear acrylamide (Ambion) and GlycoBlue (Ambion) as carriers. ChIP enrichment was analyzed by qPCR using the Mx3000P qPCR System (Stratagene). Primers used for ChIP-qPCR are listed in Supplementary Table 1.

### Electromobility shift assay (EMSA)

PAX7-FLAG constructs were expressed using a baculovirus system. Recombinant baculoviruses were generated using the Bac-to-Bac Baculovirus Expression System (Life Technologies) and used to infect Sf9 cells. Protein lysates from infected Sf9 cells were incubated with anti-FLAG M2 magnetic beads (Sigma-Aldrich) at 4 °C for 2 h. Beads were washed, and then eluted twice, first using a solution of 0.1 M glycine, and second using 100 µg/ml of FLAG peptide (Sigma-Aldrich). Eluted fractions were combined and buffer exchange to TBS was performed using Amicon Ultra-4 Centrifugal Filter Concentrator with 10 kDa nominal molecular weight limit (Millipore). For each reaction, 0.5 µg of purified PAX7-FLAG protein was used. Single-stranded probes (+) (3 µl of 10 µM) (Integrated DNA Technologies) were radiolabeled by incubation with T4 Kinase (Invitrogen) in the presence of 3 µl γ-[^32^P]-ATP for 1 h at 37 °C. Labeling was stopped by addition of STE buffer (40 mM Tris-HCl pH 8.0, 40 mM NaCl, 0.8 mM EDTA). Double-stranded probes were generated by hybridizing with opposite strand probe (−), boiling for 2 min, and slowly cooling to room temperature over 2 h. Unincorporated [^32^P]-ATP was removed by purification through Microspin G50 columns (GE Healthcare). 0.5 µg of baculovirus-purified PAX7-FLAG was incubated with 1.25 ng DNA probe and nonspecific carrier (poly dI-dC) in a binding buffer containing 75 mM NaCl, 1 mM EDTA, 1 mM DTT, 10 mM Tris-HCl pH 7.5, 6% glycerol, and 0.25% BSA at room temperature for 20 minutes. 200 ng of cold probe was used for competitive binding assays. Super shifts were carried out with the addition of 1.5 µg of anti-PAX7 antibody (Santa Cruz). Reactions were analyzed on a 5% non-denaturing polyacrylamide gel (0.5× TBE), dried onto 3 M Whatman paper and exposed to film. Probe sequences are listed in Supplementary Table 1. Uncropped scans are available in Supplementary Fig. 11.

### Co-immunoprecipitation

Indicated plasmids were transfected using Lipofectamine 2000 (Life Technologies). Cells were collected 48 h post-transfection and lysed in Triton lysis buffer (50 mM Tris pH 7.5, 150 mM NaCl, 2 mM MgCl_2_, 0.5 mM EDTA, 0.5% Triton X-100, and protease inhibitors) for 30 min on ice. Cell lysates were cleared by centrifugation and incubated with the primary antibodies overnight at 4 °C, followed by incubation with Protein A/G magnetic beads (Millipore) for 2 h at 4 °C. Beads were washed 3 times with Triton lysis buffer and eluted with Laemmli buffer. Immunoprecipitates were resolved by SDS-PAGE and analyzed by western blot with the indicated antibodies, listed in Supplementary Table 2. Uncropped scans are available in Supplementary Fig. 11 and Supplementary Fig. 12.

### Primary myoblast isolation

Primary myoblasts were purified from *C57BL/10* mice by magnetic cell separation (MACS)^60^. Briefly, dissected muscles from the hind limbs were minced and dissociated in a collagenase/dispase solution using the gentle MACS Octo Dissociator with Heaters (Miltenyi Biotec). Cells were filtered, centrifuged, and resuspended in MACS buffer (0.5% BSA, 2 mM EDTA in PBS). Satellite Cell Isolation Cocktail (Miltenyi Biotec), containing microbeads conjugated to CD11b, SCA-1, CD45 and CD31 antibodies, was added for 15 min. Cell suspensions were loaded onto a LD column (Miltenyi Biotec) in the magnetic field of a VarioMACS separator (Miltenyi Biotec) and rinsed with MACS buffer. Column flow-through (containing the lineage-negative cells) was collected and stained with anti-α7-INTEGRIN MicroBeads (Miltenyi Biotec) for 15 min. Cells were loaded onto a MS column (Miltenyi Biotec) and washed 3 times with MACS buffer. Flow-through was discarded, and the column was flushed to collect satellite cells and myoblasts. Cells were plated onto collagen-coated culture dishes.

### RT-qPCR

For siRNA transfection, primary myoblasts were transfected twice (at 0 h and 24 h) with 50 nM siMyst1 (Dharmacon) and/or 10 nM siSirt2 (IDT). Cells were collected 24 h after the second transfection. For inhibitor treatment, primary myoblasts were treated with either DMSO (control), AGK2 (10 or 20 μM) (Sigma-Aldrich) or MG149 (20 or 40 μM) (ApexBio) for 24 h. RNA was extracted using Trizol (Life Technologies). cDNA was synthesized using SuperScript III First-Strand Synthesis System (Invitrogen) following the manufacturer’s instructions. qPCR reaction was performed using iQ SYBR Green Supermix (Bio-Rad) and the Mx3000P qPCR System (Stratagene). Primers used for RT-qPCR are listed in Supplementary Table 1.

### Myofiber isolation and culture

Single myofibers were isolated from *extensor digitorum longus* (EDL) muscles of 6–8 week old mice as previously described^59^. Briefly, EDL muscles were dissected and incubated in DMEM (Gibco) containing 0.2% collagenase I (Worthington) for 1 h. Myofibers were detached by gentle trituration, washed and cultured in DMEM (Gibco) supplemented with 20% fetal bovine serum and 1% chick embryo extract. siRNA transfections were performed twice (4 h and 16 h after isolation) using Lipofectamine RNAiMAX (Life Technologies) according to the manufacturer’s instructions. siMyst1 (Dharmacon) was used at a final concentration of 50 nM, and siSirt2 (IDT) at 10 nM. Myofibers were fixed after 42 h or 72 h in culture using 4% PFA in PBS for 10 min before staining (see Immunofluoresecnce methods). Fibers from each mouse were independently treated with siRNA, and a minimum of 40 myofibers were analyzed per condition.

### Proximity ligation assay

Cultured myofibers were fixed in 4% paraformaldehyde for 10 min. Fixed myofibers were permeabilized (0.1% Triton X-100, 0.1 M Glycine, PBS) for 10 min and blocked with Duolink Blocking Solution (Sigma) for 2–3 h. Primary antibodies (chicken anti-Syndecan-4, mouse anti-Pax7, rabbit anti-acetyl-lysine, listed in Supplementary Table 2) were added overnight at 4 °C. PLA reactions were subsequently carried out using Duolink PLA plus and minus probes for mouse and rabbit (respectively) and Duolink In Situ Detection Reagents Red (Sigma) following the manufacturer’s protocol. Anti-chicken Alexa Fluor 488 secondary antibody was used simultaneously during the Duolink amplification step to label Syndecan-4. Following the manufacturer’s wash instructions, myofibers were mounted on glass slides using VECTASHIELD Hardset Antifade Mounting Medium with DAPI (Vector Laboratories). Z-stack images for each satellite cell were captured using an epifluorescent microscope with a motorized stage (Zeiss AxioObserver Z1). Deconvolution of each image was performed using Zen software (Zeiss). A max intensity Z-projection for each image was created using Fiji software. PLA interactions within the nuclei were quantified by counting distinct foci that overlapped DAPI staining using the “Find Maxima” process within Fiji software.

### Immunofluorescence

Fixed myofibers were permeabilized (0.1% Triton X-100, 0.1 M Glycine in PBS) for 10 min and blocked (5% horse serum, 2% BSA, 0.1% Triton X-100 in PBS) for 2 h. Primary antibodies (listed in Supplementary Table 2) were diluted in blocking buffer and added overnight at 4 °C. Myofibers were washed with PBS and incubated with Alexa Fluor-conjugated secondary antibodies (Life Technologies) for 1 h at room temperature. Myofibers were washed with PBS, stained with DAPI for 5 min, washed again with PBS and mounted onto glass slides using PermaFluor (Fisher).

### Flow cytometry assays

To induce muscle injury, mice were anesthetized with isoflurane, and cardiotoxin (CTX) from *Naja pallida* (10 μM, Latoxan Laboratory) was injected directly into the *tibialis anterior* (TA) muscle (40 μl) as well as into the *gastrocnemius* muscle (80 μl). CTX was injected once, and mice were euthanized 5 days after CTX injection. 3 h before sacrifice, mice were injected intraperitoneally with EdU (Invitrogen) (10 mM in PBS, 10 μl/g body weight). Hindlimb muscles were dissected, minced, and dissociated in a collagenase/dispase solution using the gentle MACS Octo Dissociator with Heaters (Miltenyi Biotec). Cells were filtered, centrifuged and resuspended in FACS buffer (10%FBS, 3 mM EDTA in PBS). For proliferation assays, cells were stained with α7-INTEGRIN and lineage markers (CD31, CD11b, SCA-1 and CD45) (listed in Supplementary Table 2), then fixed using BD Cytofix/Cytoperm Fixation/Permeabilization kit (BD Biosciences). EdU was then visualized using Click-iT EdU Imaging Kit (Invitrogen) according to the manufacturer’s instructions. Cells were then incubated with propidium iodide (20 μg/ml) and RNAse A (0.5 μg/ml) for 16 h before analysis. Analysis was performed on a BD LSRFortessa instrument using the DIVA software.

### Muscle regeneration assays

To induce muscle injury, mice were anesthetized with isoflurane, and cardiotoxin (CTX) from *Naja pallida* (50 μl of 10 μM, Latoxan Laboratory) was injected directly into the *tibialis anterior* (TA) muscle. CTX was injected once, or every 21 days for a total of three injections. Mice were euthanized either 21 days after CTX injection, or 21 days after the third injection. TA muscles were dissected and embedded within Tissue-Tek OCT (Optimal Cutting Temperature compound, Sakura). Satellite cell number, fiber number, and minimum Feret’s fiber diameter were measured by immunohistochemistry on TA sections. For minimum Feret’s fiber diameter, analysis was performed using MATLAB SMASH (semi-automatic muscle analysis using segmentation of histology) application^61^. Other counting and quantifications were performed using ImageJ software.

### Immunohistochemistry

TA muscle cryosections were fixed using 4% PFA/PBS for 2 min and permeabilized (0.1% Triton X-100, 0.1 M Glycine, PBS) for 5 min. Cryosections were washed with PBS and blocked in 5% goat serum, 2% BSA in PBS for 2 h, supplemented by M.O.M. blocking reagent (1:40, Vector Laboratories) when mouse primary antibodies were used. Primary antibodies (listed in Supplementary Table 2) were incubated overnight at 4 °C. Alexa Fluor-conjugated secondary antibodies (Life Technologies) were incubated for 1 h. Sections were washed with PBS and stained with DAPI for 10 min, rinsed with PBS, and mounted with PermaFluor mounting medium (Fisher).

### Cell sorting

Freshly isolated and fixed (quiescent) satellite cells were sorted from uninjured hindlimb muscles, and activated satellite cells were sorted from cardiotoxin-injured *tibialis anterior* and *gastrocnemius* muscles at 3 days post-injury. Muscles were minced in a collagenase/dispase solution and dissociated into a single-cell suspension using the GentleMACS Octo Dissociator with Heaters (Miltenyi Biotec). Cells were filtered, centrifuged and resuspended in FACS buffer (10%FBS, 3 mM EDTA in PBS). Freshly isolated and fixed (quiescent) satellite cells (α7-INTEGRIN^+^, CD34^+^, CD31^−^, CD11b^−^, SCA-1^−^ and CD45^−^), as well as activated satellite cells (α7-INTEGRIN^+^, VCAM1^+^, CD31^−^, CD11b^−^, SCA-1^−^ and CD45^−^), were sorted using a MoFlo XDP cell sorter (Beckman Coulter). Fixed (quiescent) satellite cells were isolated according to Machado et al.^33^. Briefly, muscles were fixed in 0.5% PFA immediately after dissection and incubated in PFA for 1 h at 4 °C. Muscle preparations were washed extensively using cold PBS, before dissociation in the collagenase/dispase solution for 40 min using the GentleMACS Octo Dissociator with Heaters (Miltenyi Biotec). FACS antibodies are listed in Supplementary Table 2.

### Biomark HD analysis

Total RNA was purified using Arcturus PicoPure RNA Isolation Kit (Applied Biosystems) using the manufacturer’s instructions. For each biological sample, 7 ng of RNA was used to synthesize cDNA using Single Cell-to-CT kit (Life Technologies) in a final volume of 5 μl. Pre-amplification was performed according to the manufacturer’s instructions using a pool of 96 Taqman primer pairs (Applied Biosystems). Samples were loaded in technical duplicates on a 96.96 Dynamic Array integrated fluidic circuit (IFC) plate together with the Taqman assays according to the manufacturer’s instructions, and qPCR reaction was performed on the Biomark HD system (Fluidigm). Fluidigm Real-Time PCR Analysis software was initially used to export raw values for downstream analysis. Gene expression was analyzed and visualized with the R-package “HTqPCR”^62^. Normalization was performed with the “deltaCt” method using reference genes: *Rps16, Rps18, Rps20, B2M, Gapdh,* and *Tbp*. Volcano plots were visualized with the R-package “EnhancedVolcano”. Fold changes were calculated with normalized values and significance conducted using student’s *t*-test.

### RNA-sequencing

Total RNA was purified using Arcturus PicoPure RNA Isolation Kit (Applied Biosystems) using the manufacturer’s instructions. For each biological sample, 15 ng of RNA was used as input for the NEBNext Ultra II Directional RNA Library Prep Kit (New England Biolabs). The libraries were sequenced using a NextSeq 500 Mid Output 2X150bp cycle kit (Illumina). Transcripts were quantified with salmon v.1.3.0^63^ against an index built from the GENCODE vM25 assembly with inclusion of genomic decoy sequences. Data were loaded into R using the tximport library^64^, and the gene/count matrix was filtered to retain only genes with five or more mapped reads in two or more samples. Differential expression was assessed using DESeq2 v1.28.1;^65^ expression differences between *Pax7*^*KR*^ *versus Pax7*^*WT*^ replicates were calculated using the DESeq2 results() function. Lists of significantly changed genes were identified using a *q*-value (Benjamini–Hochberg corrected *p*-value) cutoff of 0.10.

#### Annotation with PAX7 binding sites

PAX7 binding sites identified by Soleimani et al.^10^ were loaded into R and annotated using the ChIPseeker R package^66^ and the “org.Mm.eg.db” annotation database. Peak annotations, including the PAX7 peak to TSS distance, were matched to DE results using the Ensembl geneID identifier; these results were used to identify DE genes with associated PAX7 peaks, peak-TSS range, and number of peaks.

#### De novo motif analysis

PAX7 binding locations associated with sets of DE genes were extracted from the ChIPseeker annotation results. These peak locations were passed to the HOMER^67^ findMotifsGenome to identify enriched motifs within the PAX7 binding regions.

#### Gene ontology analysis

DE genes with associated PAX7 peaks and expression ≥baseMean(50) were submitted to gene ontology analysis using the online software g:Profiler^68^.

#### Gene set enrichment analysis

DESeq2 differential expression results for protein-coding genes were ranked using the metric *−log10(p-value)* * *sign(log2FoldChange)*. This ranks the gene list to place the genes with the most significant positive fold changes (higher in *Pax7*^*KR*^) at the top, and the most significant negative fold changes (lower in *Pax7*^*KR*^) at the bottom. A list of gene symbols and numeric ranks was loaded into the GSEA v4.1.0 desktop application^69^, and GSEA enrichment analysis was performed using the GSEAPreranked method against the MSigDb v7.2 Hallmark and Canonical Pathway gene sets, using the remapping from mouse gene symbols to human orthologs provided with GSEA.

### Statistics and reproducibility

No statistical method was used to predetermine sample size. All experiments were performed with at least three biological replicates as indicated in the figure legends, and results are presented as the mean ± standard error of the mean (SEM). For EMSA (Fig. 1h), co-immunoprecipitation and immunoblots (Fig. 2a–c, Fig. 2f, Supplementary Figs. 1, 2,  3a, b), experiments have been performed at least three independent times with similar results. For immunostaining (Figs. 2d, e,  4a, d,  5a, d,  6a, c,  7a, d, Supplementary Figs. 1b,  3c, d), the representativity (in % of the cells examined) and the number of biological replicates are indicated in the respective figure legends. Appropriate quantification of the phenotype is also included as a separate figure panel when relevant. Experimental design incorporated user blinding when possible. Statistical comparisons between groups were made using two-tailed Student’s *t* test (paired for biologically matched samples, and unpaired for unrelated samples) or univariate ANOVA, as appropriate. Statistical analysis was performed using GraphPad Prism. The level of significance is indicated as follows: *p ≤ 0.05, **p ≤ 0.01, ***p ≤ 0.001, (*p*-values <0.05 were considered as statistically significant).

### Reporting summary

Further information on research design is available in the Nature Research Reporting Summary linked to this article.

## Supplementary information

Reporting Summary

Supplementary Information

## Data Availability

Raw data for the RNA-sequencing have been deposited in the Gene Expression Omnibus database under the accession code GSE167532. The raw images for the immunoblots are provided in Supplementary Figs. 11 and 12. The source data underlying Figs. 1f, 1g, 2g, 3a–c, 4b, 4c, 4e, 5b, 5c, 5e, 6b, 6d–f, 7b, 7c, 7e–h and Supplementary Figs. 3e–h, 4a–c, 5a–i, 6a–c, 6f, 7a–d, 8b, 9b, 9c are provided in the Source Data file. All other datasets generated during and/or analyzed during the current study are available from the corresponding author on reasonable request. Source data are provided with this paper.

## Source data

Source Data
