# Supplementary material for: Acetylation of PAX7 controls muscle stem cell self-renewal and differentiation potential in mice
Source: Nat Commun. 2021 May 31;12:3253. doi: 10.1038/s41467-021-23577-z (PMC8167170; doi:10.1038/s41467-021-23577-z)
Supplement: Supplementary file 1 — Reporting Summary [file 41467_2021_23577_MOESM1_ESM.pdf]

nature research

Corresponding author(s): Michael Rudnicki

Last updated by author(s): May 3, 2021

# Reporting Summary

Nature Research wishes to improve the reproducibility of the work that we publish. This form provides structure for consistency and transparency in reporting. For further information on Nature Research policies, see our Editorial Policies and the Editorial Policy Checklist.

## Statistics

For all statistical analyses, confirm that the following items are present in the figure legend, table legend, main text, or Methods section.

| n/a | Confirmed | |
|---|---|---|
| ☐ | ☒ | The exact sample size (*n*) for each experimental group/condition, given as a discrete number and unit of measurement |
| ☐ | ☒ | A statement on whether measurements were taken from distinct samples or whether the same sample was measured repeatedly |
| ☐ | ☒ | The statistical test(s) used AND whether they are one- or two-sided<br>*Only common tests should be described solely by name; describe more complex techniques in the Methods section.* |
| ☒ | ☐ | A description of all covariates tested |
| ☐ | ☒ | A description of any assumptions or corrections, such as tests of normality and adjustment for multiple comparisons |
| ☒ | ☐ | A full description of the statistical parameters including central tendency (e.g. means) or other basic estimates (e.g. regression coefficient) AND variation (e.g. standard deviation) or associated estimates of uncertainty (e.g. confidence intervals) |
| ☒ | ☐ | For null hypothesis testing, the test statistic (e.g. *F*, *t*, *r*) with confidence intervals, effect sizes, degrees of freedom and *P* value noted<br>*Give P values as exact values whenever suitable.* |
| ☒ | ☐ | For Bayesian analysis, information on the choice of priors and Markov chain Monte Carlo settings |
| ☒ | ☐ | For hierarchical and complex designs, identification of the appropriate level for tests and full reporting of outcomes |
| ☒ | ☐ | Estimates of effect sizes (e.g. Cohen's *d*, Pearson's *r*), indicating how they were calculated |

*Our web collection on statistics for biologists contains articles on many of the points above.*

## Software and code

Policy information about availability of computer code

| | |
|---|---|
| Data collection | Mascot (mass spectrometry), Bio-Rad CFX96 Maestro instrument (qPCR), Zeiss Axio Observer.D1 (microscopy), Summit (FACS), BD FACSDiva software (flow cytometry), Biomark HD Data Collection Software (Biomark), NextSeq 500 Mid Output 2X150bp cycle kit (Illumina) |
| Data analysis | R software, GraphPad Prism v7.00, MATLAB-MAthWorks r2015a, MATLAB application SMASH (Semi-Automatic Muscle Analysis using Segmentation of Histology), ImageJ FIJI (imaging), Zeiss ZEN 2 software (microscopy), FlowJo v10 (flow cytometry), Fluidigm Real-Time PCR Analysis software, salmon v.1.3.0 (RNA-sequencing), DESeq2 v1.28.1 (RNA-sequencing), HOMER (RNA-sequencing), g:Profiler (RNA-sequencing), GSEA v4.1.0 (RNA-sequencing) |

For manuscripts utilizing custom algorithms or software that are central to the research but not yet described in published literature, software must be made available to editors and reviewers. We strongly encourage code deposition in a community repository (e.g. GitHub). See the Nature Research guidelines for submitting code & software for further information.

## Data

Policy information about availability of data

All manuscripts must include a data availability statement. This statement should provide the following information, where applicable:

- Accession codes, unique identifiers, or web links for publicly available datasets
- A list of figures that have associated raw data
- A description of any restrictions on data availability

The RNA-sequencing has been deposited in the Gene Expression Omnibus under the accession code GSE167532. The raw images for the immunoblots are provided in Supplementary Figures 11 and 12. The source data underlying Figures 1f, 1g, 2g, 3a-c, 4b, 4c, 4e, 5b, 5c, 5e, 6b, 6d-f, 7b, 7c, 7e-h and Supplementary Figures 3e-h, 4a-c, 5a-i, 6a-c, 6f, 7a-d, 8b, 9b, 9c are provided in the Source Data file. All other datasets generated during and/or analyzed during the current study are available from the corresponding author on reasonable request.

# Field-specific reporting

Please select the one below that is the best fit for your research. If you are not sure, read the appropriate sections before making your selection.

- [x] Life sciences
- [ ] Behavioural & social sciences
- [ ] Ecological, evolutionary & environmental sciences

For a reference copy of the document with all sections, see nature.com/documents/nr-reporting-summary-flat.pdf

# Life sciences study design

All studies must disclose on these points even when the disclosure is negative.

| | |
|---|---|
| Sample size | Sample size is indicated in the figure legends for each experiment. No sample size calculation was performed. Sample size was determined based on the magnitude and consistency of measurable differences between groups. |
| Data exclusions | No data were excluded from the analysis. |
| Replication | Biological and independent replicate experiments were successful. They were replicated independently by different co-authors. |
| Randomization | Pax7-KR and Pax7-WT mice were allocated to muscle regeneration experiments and fiber isolation based on genotype and irrespective of sex. There were no other selection criteria for the allocated animals. Other experiments did not require randomization. |
| Blinding | The researchers were blinded to allocation during analysis and outcome assessment. Animal experiments were blinded when possible. |

# Reporting for specific materials, systems and methods

We require information from authors about some types of materials, experimental systems and methods used in many studies. Here, indicate whether each material, system or method listed is relevant to your study. If you are not sure if a list item applies to your research, read the appropriate section before selecting a response.

## Materials & experimental systems

| n/a | Involved in the study | |
|---|---|---|
| [ ] | [x] | Antibodies |
| [ ] | [x] | Eukaryotic cell lines |
| [x] | [ ] | Palaeontology and archaeology |
| [ ] | [x] | Animals and other organisms |
| [x] | [ ] | Human research participants |
| [x] | [ ] | Clinical data |
| [x] | [ ] | Dual use research of concern |

## Methods

| n/a | Involved in the study | |
|---|---|---|
| [x] | [ ] | ChIP-seq |
| [ ] | [x] | Flow cytometry |
| [x] | [ ] | MRI-based neuroimaging |

## Antibodies

| | |
|---|---|
| Antibodies used | The list of antibodies can be found in the manuscript, in Supplementary Table 2. |
| Validation | All the antibodies were validated in previous published work from the lab and by the manufacturers listed in the Supplementary Table 2. |

## Eukaryotic cell lines

Policy information about cell lines

| | |
|---|---|
| Cell line source(s) | C2C12, CoS-7 and Sf9 cells were purchased from ATCC. |
| Authentication | C2C12, COS-7 and Sf9 cells were authenticated by ATCC. |
| Mycoplasma contamination | The cells were not contaminated by mycoplasma as determined by the MycoSensor PCR Assay Kit (Agilent Technologies). |
| Commonly misidentified lines (See ICLAC register) | N/A |

## Animals and other organisms

Policy information about studies involving animals; ARRIVE guidelines recommended for reporting animal research

| | |
|---|---|
| Laboratory animals | Information about species, strain, sex and age of the mice used can be found in the "Methods" section of the manuscript (Animal models). |
| Wild animals | N/A |
| Field-collected samples | N/A |
| Ethics oversight | All experimental protocols for mice used in this study were performed in accordance with the guidelines established by the University of Ottawa Animal Care Committee, which is based on the guidelines of the Canadian Council on Animal Care (CCAC). Protocols were approved by the Animal Research Ethics Board (AREB) at the University of Ottawa. |

Note that full information on the approval of the study protocol must also be provided in the manuscript.

## Flow Cytometry

### Plots

Confirm that:

- [x] The axis labels state the marker and fluorochrome used (e.g. CD4-FITC).
- [x] The axis scales are clearly visible. Include numbers along axes only for bottom left plot of group (a 'group' is an analysis of identical markers).
- [x] All plots are contour plots with outliers or pseudocolor plots.
- [x] A numerical value for number of cells or percentage (with statistics) is provided.

### Methodology

| | |
|---|---|
| Sample preparation | Sample preparation for flow cytometry analysis can be found in the "Methods" section of the manuscript (Flow cytometry assays). Sample preparation for cell sorting can be found in the "Methods" section of the manuscript (Cell sorting and Biomark analysis). Single cell suspensions were obtained from hindlimb muscles. Cell doublets were excluded from the analysis (FSC-H vs FSC-A or FSC-W vs FSC-A). Satellite cells (CD31-, CD45-, SCA1-, CD11b-, Itga7+, CD34+/VCAM+) were sorted and used for analysis. |
| Instrument | Flow cytometry analyses were performed on a BD LSRFortessa cell analyzer and FACS was performed on MoFlo XDP at the Ottawa Hospital Research Institute. |
| Software | Flow cytometry data acquisition and analysis: FACSDiva<br>FACS data acquisition: Summit |
| Cell population abundance | Purity of the post-sort fractions was determined by flow cytometry on the sorted samples. Only samples that were >90% pure were kept for analysis. |
| Gating strategy | Gating strategy was performed using unstained and single-stained samples. The gating strategy for sorting the satellite cells is provided in Supplementary Figure 10. |

- [x] Tick this box to confirm that a figure exemplifying the gating strategy is provided in the Supplementary Information.
