# Supplementary material for: Acetylation of PAX7 controls muscle stem cell self-renewal and differentiation potential in mice
Source: Nat Commun. 2021 May 31;12:3253. doi: 10.1038/s41467-021-23577-z (PMC8167170; doi:10.1038/s41467-021-23577-z)
Supplement: Supplementary file 2 — Supplementary Information [file 41467_2021_23577_MOESM2_ESM.pdf]

SUPPLEMENTARY INFORMATION

Acetylation of PAX7 Controls Muscle Stem Cell Self-Renewal and Differentiation Potential in Mice

Marie-Claude Sincennes[1,2], Caroline E. Brun[1,2], Alexander Y.T. Lin[1,2],Tabitha Rosembert[1,2], David Datzkiw[1,2], John Saber[1,2], Hong Ming[1,2], Yoh-ichi Kawabe[1,2], Michael A. Rudnicki[1,2*]

1. Sprott Centre for Stem Cell Research
   Regenerative Medicine Program
   Ottawa Hospital Research Institute
   Ottawa, ON
   K1H 8L6, Canada

2. Department of Cellular and Molecular Medicine
   Faculty of Medicine
   University of Ottawa
   Ottawa, ON
   K1H 8M5, Canada

* Corresponding Author:
Email: mrudnicki@ohri.ca
Tel: (613) 739-6740
Fax: (613) 739-6294

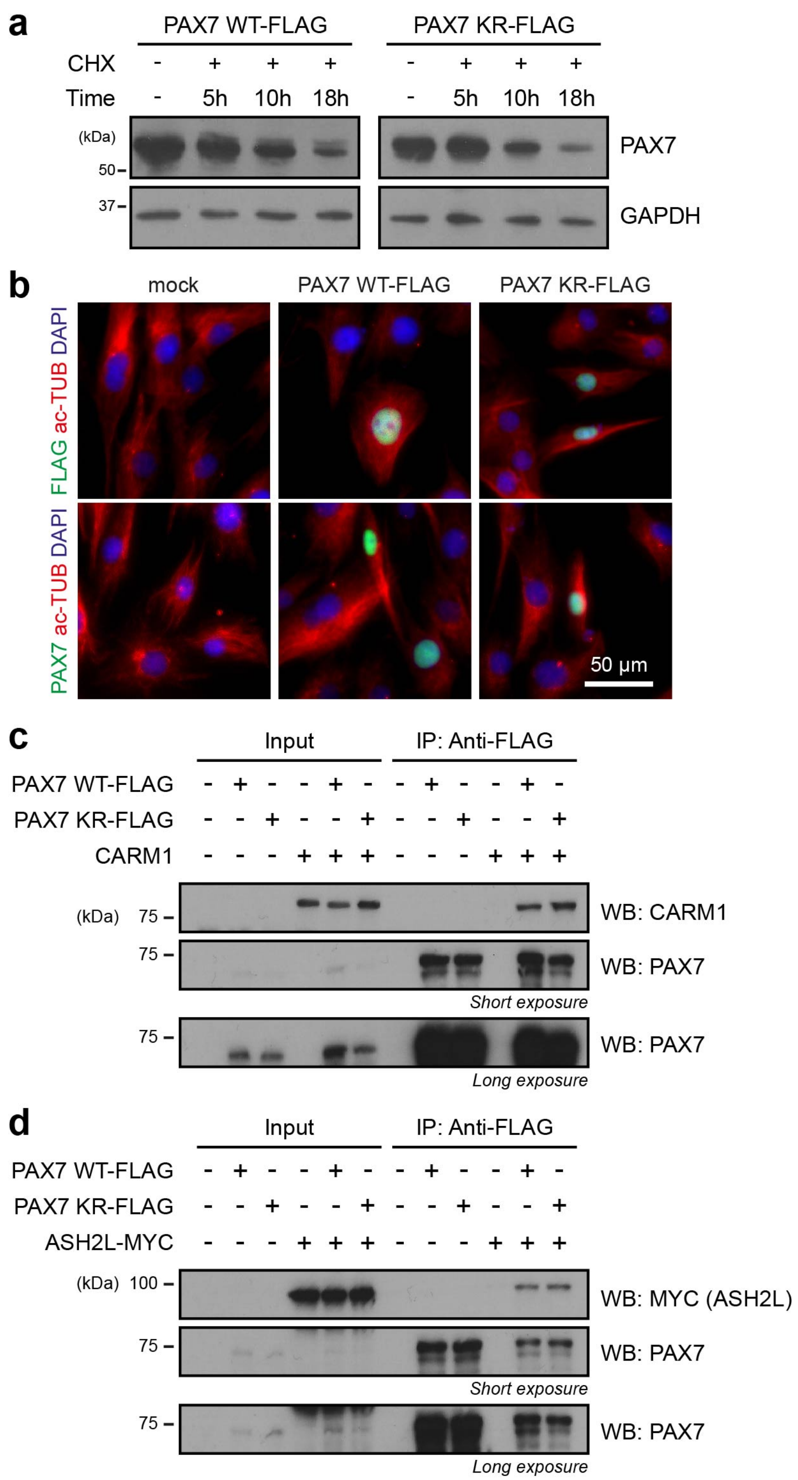

Supplementary Figure 1. Acetylation of PAX7 does not regulate its protein stability and function, or its nuclear localization

a) C2C12 myoblasts were transfected with plasmids encoding either WT (PAX7 WT-FLAG) or K105/193R PAX7 (PAX7 KR-FLAG). 48h post-transfection, cells were treated with cycloheximide (100μg/ml) for the indicated times. Protein expression was detected by immunoblotting using the indicated antibodies.

b) C2C12 myoblasts transfected as in (**a**) were fixed and stained for FLAG (green, upper panel) or PAX7 (green, lower panel) and acetylated α-TUBULIN (ac-TUB, red), and nuclei were counterstained with DAPI. Scale bar represents 50μm. Images are representative of ≥ 90% of the cells examined (n = 3 independent experiments).

c) Cells were transfected with plasmids encoding FLAG-tagged PAX7 WT or KR, together with MYC-tagged *Carm1* plasmid DNA. The interaction between PAX7 and CARM1 was determined by immunoprecipitation using anti-FLAG antibodies.

d) Cells were transfected with plasmids encoding FLAG-tagged PAX7 WT or KR, together with MYC-tagged *Ash2l* plasmid DNA, as indicated. The interaction between PAX7 and ASH2L was determined by immunoprecipitation using anti-FLAG antibodies.

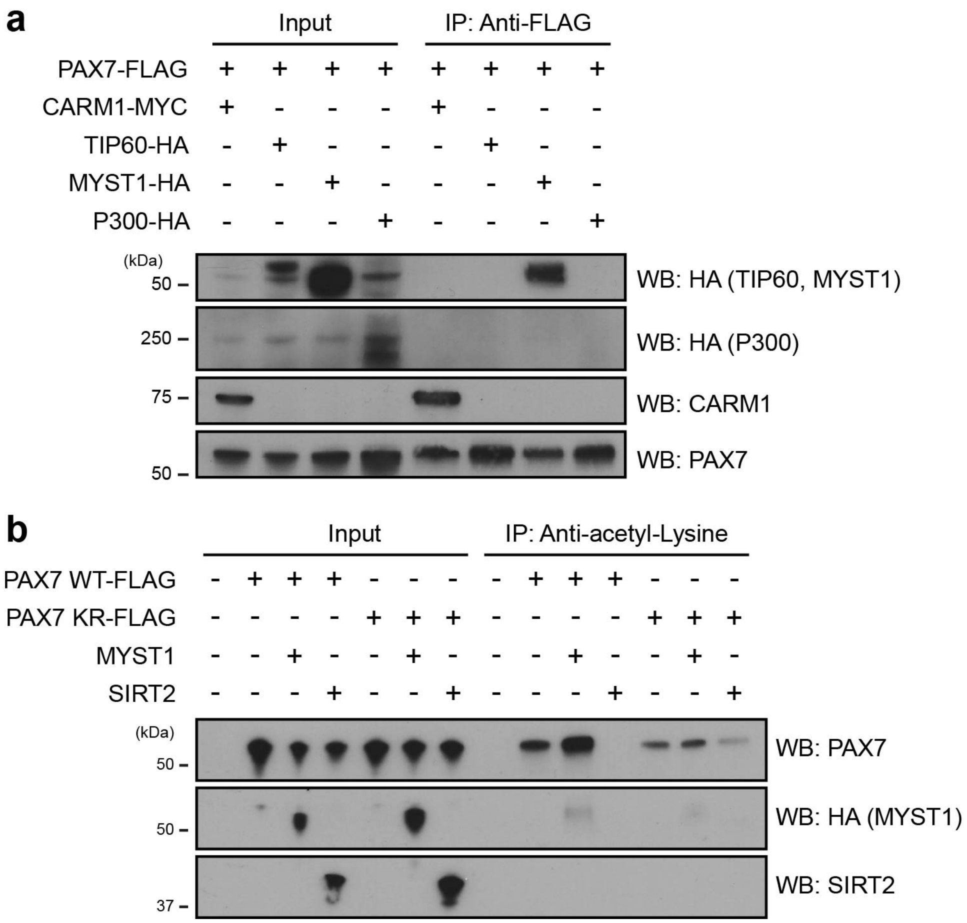

Supplementary Figure 2. PAX7 specifically interacts with the acetyltransferase MYST1 to regulate its acetylation status

a) C2C12 myoblasts were transfected with plasmids encoding FLAG-PAX7, MYC-CARM1 and the different HA-tagged acetyltransferases (HAT), as indicated. The interaction between PAX7 and the different HATs was determined by immunoprecipitation using anti-FLAG antibodies. CARM1 was used as a positive control for PAX7-interacting protein.

b) Cells were transfected with plasmids encoding PAX7 (WT or K105/193R, KR), MYST1 and SIRT2, and lysates were subjected to immunoprecipitation using anti-acetyl-lysine antibodies. Immunoblotting was performed with the indicated antibodies.

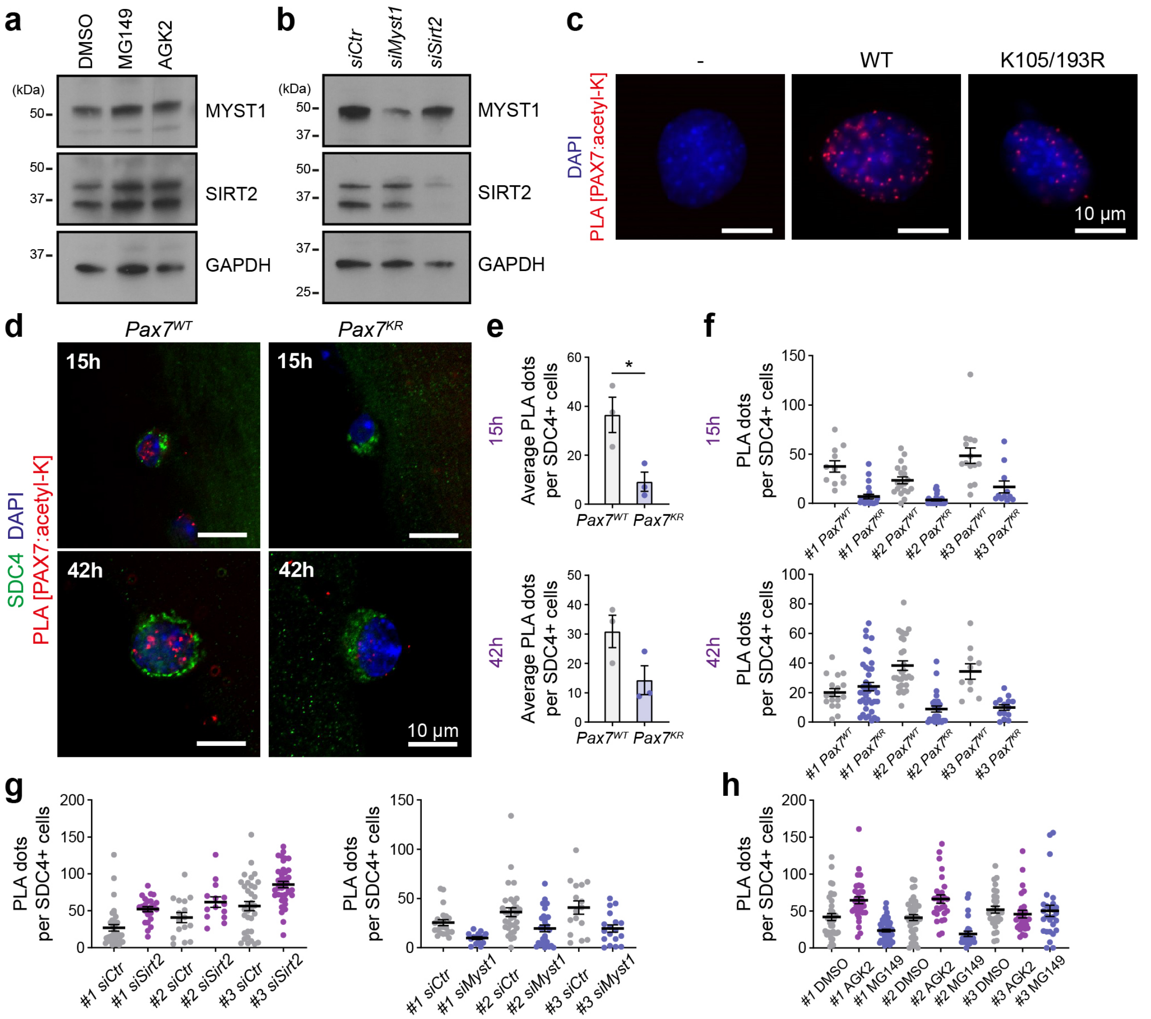

Supplementary Figure 3. PAX7 is acetylated in satellite cells

a, b) Expression level of MYST1 and SIRT2 in primary myoblasts following siRNA and drug treatments. **a**) Primary myoblasts were collected after 24h-treatment with the SIRT2 inhibitor AGK2 (20µM) and the MYST1 inhibitor MG149 (40µM). **b**) Primary myoblasts were transfected twice with siRNAs targeting *Myst1* or *Sirt2*, and collected 24h after the second transfection. Protein expression was detected by immunoblotting using the indicated antibodies, and GAPDH was used as a loading control.

c) Representative PAX7:acetylated-lysine PLA (red) performed on C2C12 myoblasts, transfected with plasmids encoding PAX7 (WT or K105/193R, KR) as indicated. Nuclei are counterstained with DAPI (blue). Scale bar represents 10 μm. Images are representative of ≥ 80% of the cells examined (n = 3 independent experiments).

d) Representative PAX7:acetylated-lysine PLA (red) performed on satellite cells from $Pax7^{WT}$ and $Pax7^{KR}$ cultured myofibers for the indicated times. Satellite cells are marked by expression of Syndecan-4 (green) and nuclei are counterstained with DAPI (blue). Scale bar represents 10 μm. Images are representative of ≥ 75% of the myofibers examined (n = 3 mice).

e) Quantification of the PLA signals from **d**), represented as the mean ± SEM. Quantification was obtained by counting the number of nuclear PLA puncta for each satellite cell (time 15h: n = 44 $Pax7^{WT}$, n = 62 $Pax7^{KR}$ cells from 3 mice) (time 42h: n = 56 $Pax7^{WT}$, n = 79 $Pax7^{KR}$ cells from 3 mice) (Two-tailed unpaired t test: *p = 0,0291).

f-h) Quantification of PLA signal for each individual cell analyzed in three biological replicates. Data are presented as mean values ± SEM **f**) Corresponds to Supplementary Figure 3e; **g**) corresponds to Figure 4 b,c; **h**) corresponds to Figure 4e.

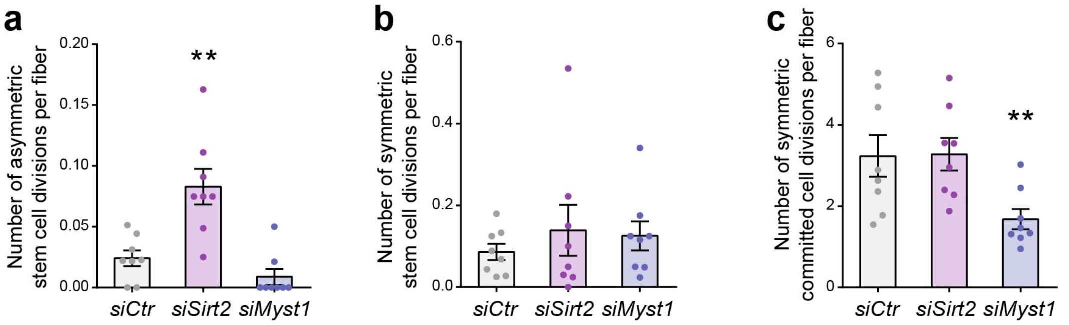

Supplementary Figure 4. MYST1 and SIRT2 regulate satellite cell self-renewal

a, b) Absolute number of (**a**) asymmetric ($YFP^{–}/YFP^{+}$) and (**b**) symmetric ($YFP^{–}/YFP^{–}$) satellite stem cell divisions per muscle fiber, after transfection with siRNAs against *Myst1* and *Sirt2.* Data are presented as mean values ± SEM (n = 8 samples from independent mice) (One-way ANOVA uncorrected Fisher's LSD test: **p = 0,0038).

c) Absolute number of committed (YFP^{+}/YFP^{+}) satellite cell divisions per muscle fiber, after *Myst1* and *Sirt2* knockdown. Data are presented as mean values ± SEM (n = 8 samples from independent mice) (One-way ANOVA uncorrected Fisher's LSD test: **p = 0,0033).

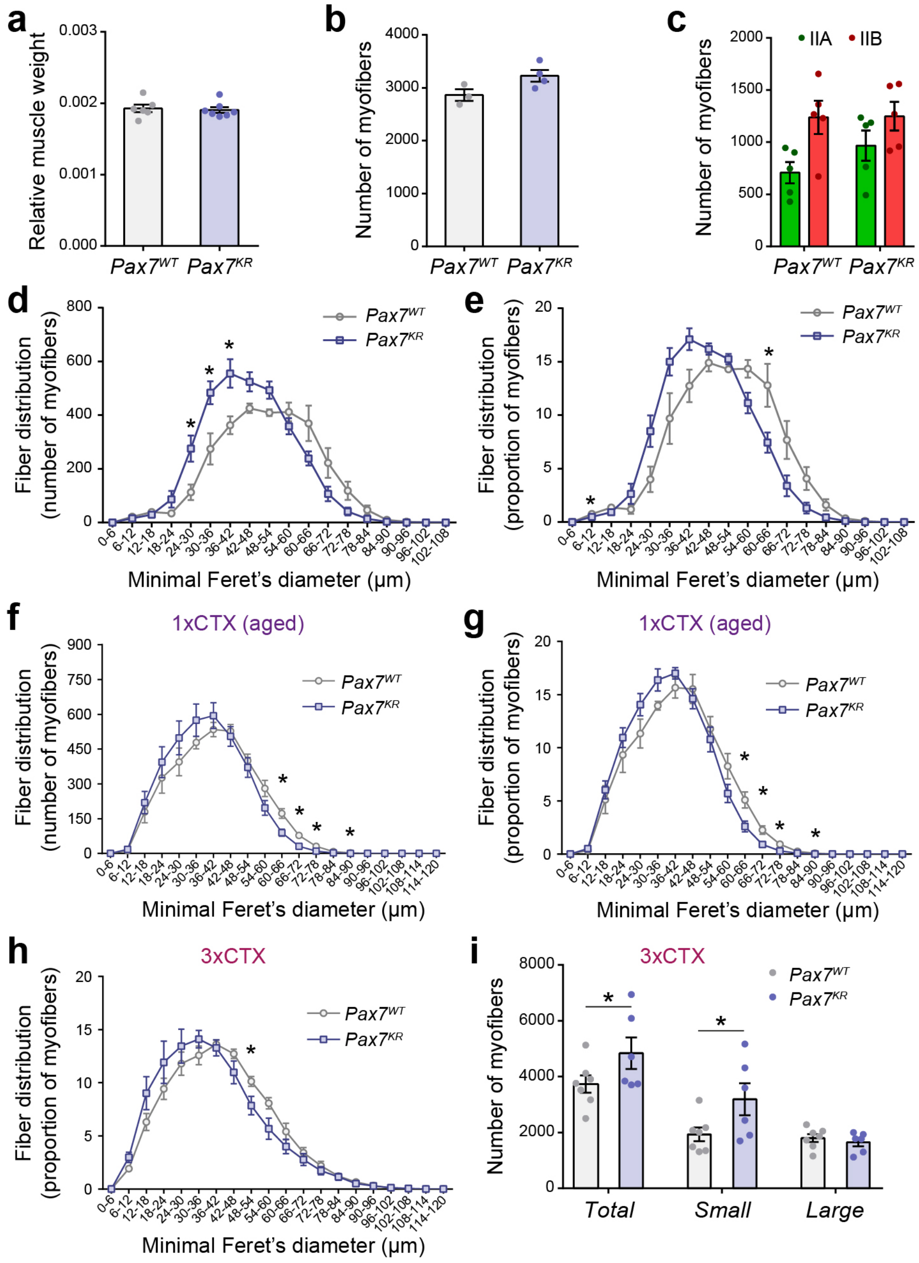

Supplementary Figure 5. ***Pax7*^{KR} mice display smaller muscle fibers**

a) Weight of uninjured TA muscle relative to body weight. Data are presented as mean values ± SEM (n = 6 mice per group).

b) Number of muscle fibers per uninjured TA muscle. Data are presented as mean values ± SEM (n = 3 for *Pax7*^{WT} mice, n = 4 for *Pax7*^{KR} mice).

c) Quantification of the number of type IIA and type IIB muscle fibers in uninjured TA muscles (contralateral leg). Data are presented as mean values ± SEM (n = 5 mice per group).

d, e) Minimal fiber Feret's diameter distribution of uninjured TA muscle from *Pax7*^{WT} and *Pax7*^{KR} mice, represented as (**e**) absolute number of muscle fibers or as (**f**) percentage of muscle fibers. Data are presented as mean values ± SEM (n = 3 for *Pax7*^{WT} mice, n = 4 for *Pax7*^{KR} mice) (Multiple unpaired t tests: *p = 0,048; *p = 0,031; *p =0,037 respectively in (**d**); *p = 0,035; *p = 0,046 respectively in (**e**)).

f, g) Minimal fiber Feret's diameter distribution of TA muscle from aged *Pax7*^{WT} and *Pax7*^{KR} mice, 21 days following one CTX-induced muscle injury, represented as (**f**) absolute number of muscle fibers or as (**g**) percentage of muscle fibers. Data are presented as mean values ± SEM (n = 4 for *Pax7*^{WT} mice, n = 5 for *Pax7*^{KR} mice) (Multiple unpaired t tests: *p = 0,015; *p = 0,012; *p = 0,032; *p = 0,040 respectively in (**f**); *p = 0,024; *p = 0,015; *p = 0,041; *p = 0,036 respectively in (**g**)).

h) Minimal fiber Feret's diameter of injured TA muscles from *Pax7*^{WT} and *Pax7*^{KR} mice after 3 consecutive rounds of CTX injury, represented as percentage of muscle fibers. Data are presented as mean values ± SEM (n = 6 mice per group) (Multiple unpaired t tests: *p = 0,021).

i) Number of total, large and small muscle fibers per TA muscle after triple CTX injury. Data are presented as mean values ± SEM (n = 6 mice per group) (Two-way ANOVA uncorrected Fisher's LSD test: *p = 0,0359 in Total; *p = 0,0186 in Small).

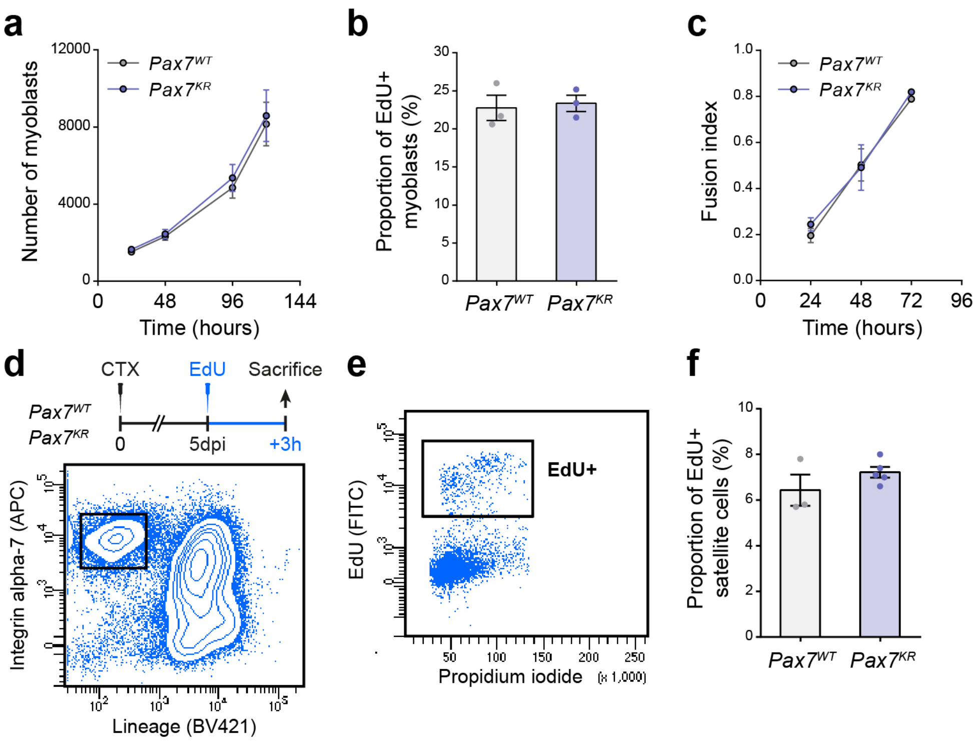

Supplementary Figure 6. ***Pax7^{KR}*** **satellite cells proliferate normally**

a) Primary myoblasts were isolated from *Pax7^{WT}* and *Pax7^{KR}* mice and seeded at time 0 at the same cell concentration. Myoblasts were cultured for 5 days in growth conditions and their numbers were quantified at each indicated time point. Data are presented as mean values ± SEM (n = 3 biologically independent samples).

b) Primary myoblasts were treated with EdU for 2h, fixed, and analyzed for EdU incorporation by flow cytometry. Data are presented as mean values ± SEM (n = 3 biologically independent samples).

c) Primary myoblasts were plated at high confluency and induced to differentiate for the indicated time points. Myotubes were stained using anti-myosin heavy chain antibody and fusion index was quantified. Data are presented as mean values ± SEM (n = 3 biologically independent samples).

d) Representative flow cytometry plot of muscle cells at 5 days post-CTX injury. Mice were injected with cardiotoxin in the *tibialis anterior* (TA) muscle to induce satellite cell activation and proliferation. At 5 days post-cardiotoxin (CTX) injury, mice were injected intraperitoneally with EdU and analyzed 3h later. Muscle cells were fixed and analyzed for cell proliferation using EdU content. Satellite cells ($CD31^-$, $SCA1^-$, $CD11b^-$, $CD45^-$, α7-$INTEGRIN^+$) were considered for analysis in panels **e,f**.

e) Representative flow cytometry plot of EdU-treated satellite cells. Satellite cells were fixed and stained for EdU (proliferating cells) and propidium idodide (DNA content).

f) Quantification of the proportion of EdU-positive satellite cells at 5 days post-CTX injury. Data are presented as mean values ± SEM (n = 3 for $Pax7^{WT}$ mice, n = 5 for $Pax7^{KR}$ mice).

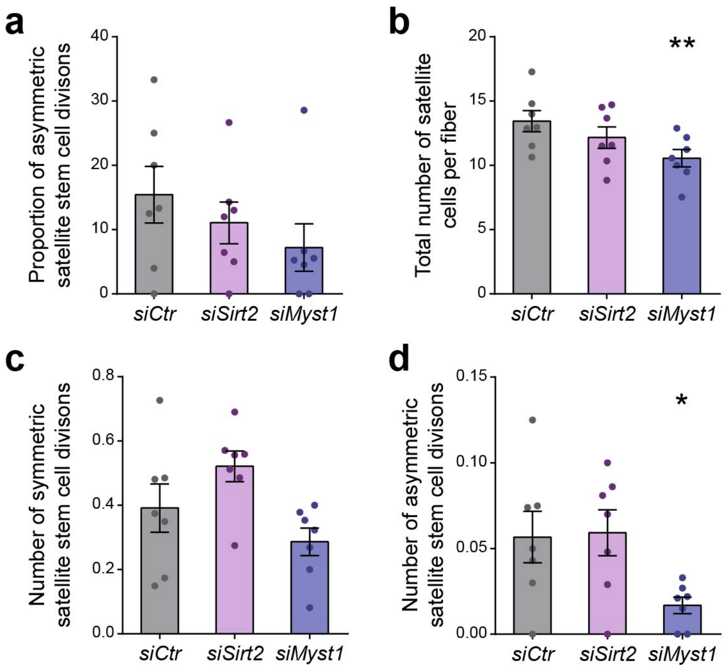

Supplementary Figure 7. MYST1 and SIRT2 do not regulate satellite cell self-renewal in *Pax7*^{KR} mice

a) Proportion of asymmetric satellite stem cell division on *Pax7*^{KR}*:Myf5-Cre:R26R-EYFP* myofibers cultured for 42h. Myofibers were transfected with siRNA against *Sirt2* or *Myst1* as indicated. Data are presented as mean values ± SEM (n = 7 samples from independent *Pax7*^{KR} mice).

b) Number of satellite cells on myofibers cultured for 42h and transfected as in (**a**). Data are presented as mean values ± SEM (n = 7 samples from independent *Pax7*^{KR} mice) (One-way ANOVA uncorrected Fisher's LSD test: **p = 0,003).

c, d) Absolute numbers of (**c**) symmetric (YFP-/YFP-) and (**d**) asymmetric (YFP+/YFP-) satellite cell doublets on myofibers cultured for 42h and transfected as in (**a**). Data are presented as mean values ± SEM (n = 7 samples from independent *Pax7*^{KR} mice) (One-way ANOVA uncorrected Fisher's LSD test: *p = 0,0299).

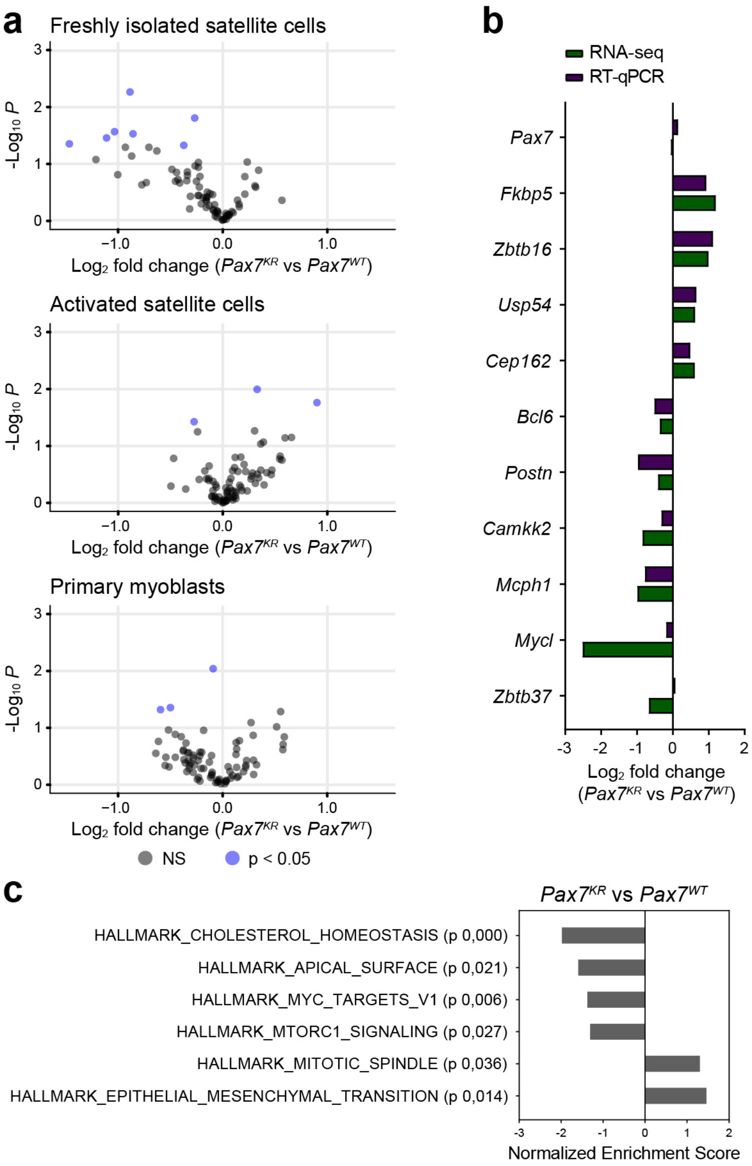

Supplementary Figure 8. PAX7 acetylation regulates target gene expression in freshly isolated satellite cells

a) Volcano plot representing the difference in gene expression for 80 PAX7 target genes between *Pax7*^{WT} and *Pax7*^{KR} freshly isolated satellite cells (FISC), activated satellite cells (ASC) and primary myoblasts, determined by Biomark.

b) Expression level of differentially expressed genes between *Pax7*^{WT} and P*ax7*^{KR} satellite cells. Gene expression was determined by RT-qPCR (normalized to *Rps18* expression). Data are presented as log2-transformed mean values (n = 4 biologically independent samples for each genotype).

c) Summary of Gene set enrichment analysis (GSEA) normalized enrichment scores of *Pax7*^{KR} FISCs compared to *Pax7*^{WT} FISCs.

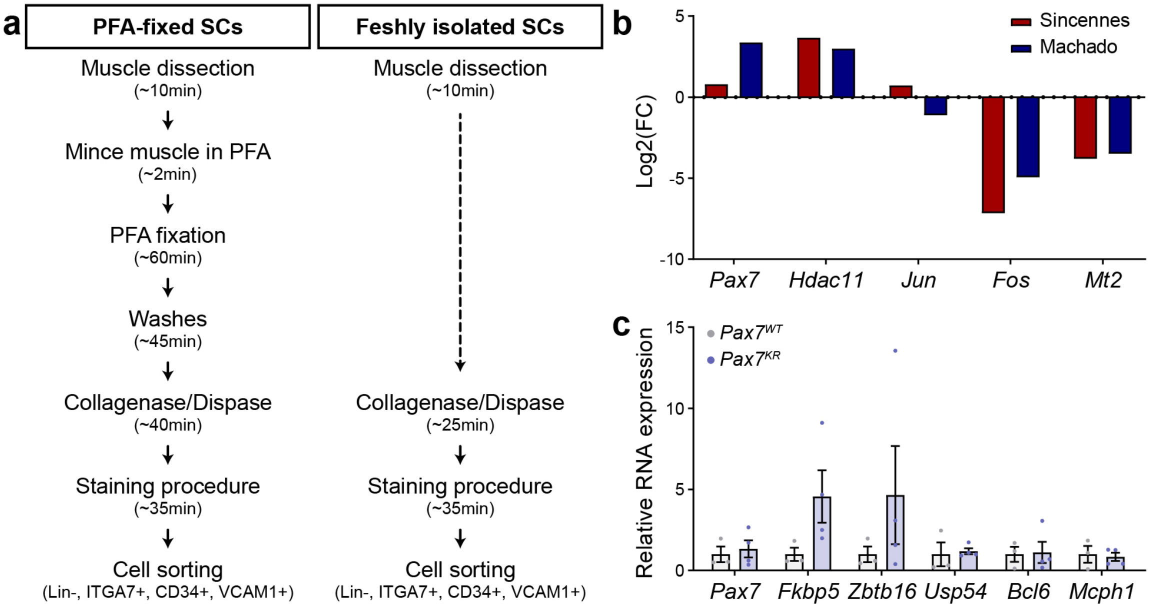

Supplementary Figure 9. PAX7 acetylation regulates a subset of target genes in quiescent satellite cells

a) Experimental procedures used to purify freshly isolated satellite cells and quiescent, PFA-fixed satellite cells.

b) Differences in gene expression between freshly isolated (FISCs) and quiescent, PFA-fixed (QSCs) satellite cells. Sincennes, red: gene expression determined by RT-qPCR (n = 6 FISCs, n = 7 QSCs), normalized to *Rps18* expression, and expressed as log2 fold change (FISCs *versus* QSCs). Machado, blue: gene expression determined by RNA-sequencing[1], expressed as log2 fold change (FISCs *versus* QSCs).

c) Gene expression analysis of $Pax7^{WT}$ and $Pax7^{KR}$ quiescent, PFA-fixed satellite cells. Gene expression was determined by RT-qPCR (normalized to *Rps18* expression). Data are presented as mean values ± SEM (n = 3 $Pax7^{WT}$, n = 4 $Pax7^{KR}$).

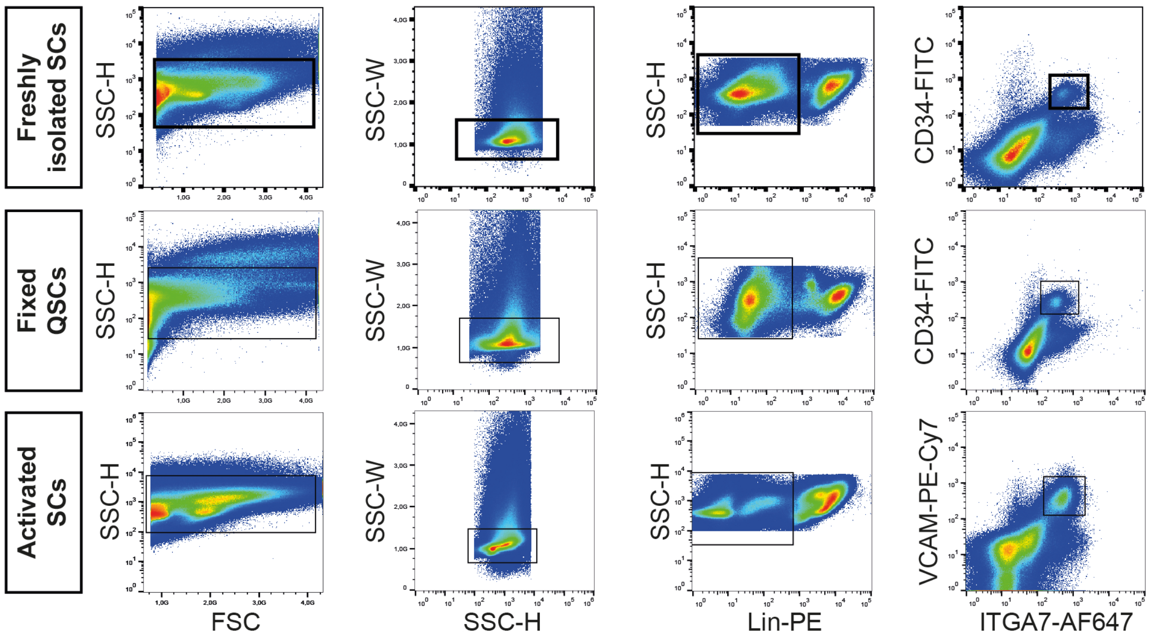

Supplementary Figure 10. Gating strategy for quiescent and activated satellite cell sort

Freshly isolated and fixed QSCs: Single cell suspensions were obtained from all hindlimb muscles. Cell doublets were excluded from the analysis (SSC-H *vs* SSC-W). Satellite cells (CD31-, CD45-, SCA1-, CD11b-, ITGA7+, CD34+) were sorted and used for RNA-seq analysis (**Figure 8**), for Biomark analysis (**Supplementary Figure 8a**) as well as for RT-qPCR analyses (**Supplementary Figure 8b**, **Supplementary Figure 9b**).

Activated SCs: Single cell suspensions were obtained from *tibialis anterior* (TA) and *gastrocnemius* (GA) muscles, 3 days post-cardiotoxin injury. Cell doublets are excluded from the analysis (SSC-H *vs* SSC-W). Satellite cells (CD31-, CD45-, SCA1-, CD11b-, ITGA7+, VCAM1+) were sorted and used for Biomark analysis (**Supplementary Figure 8a**).

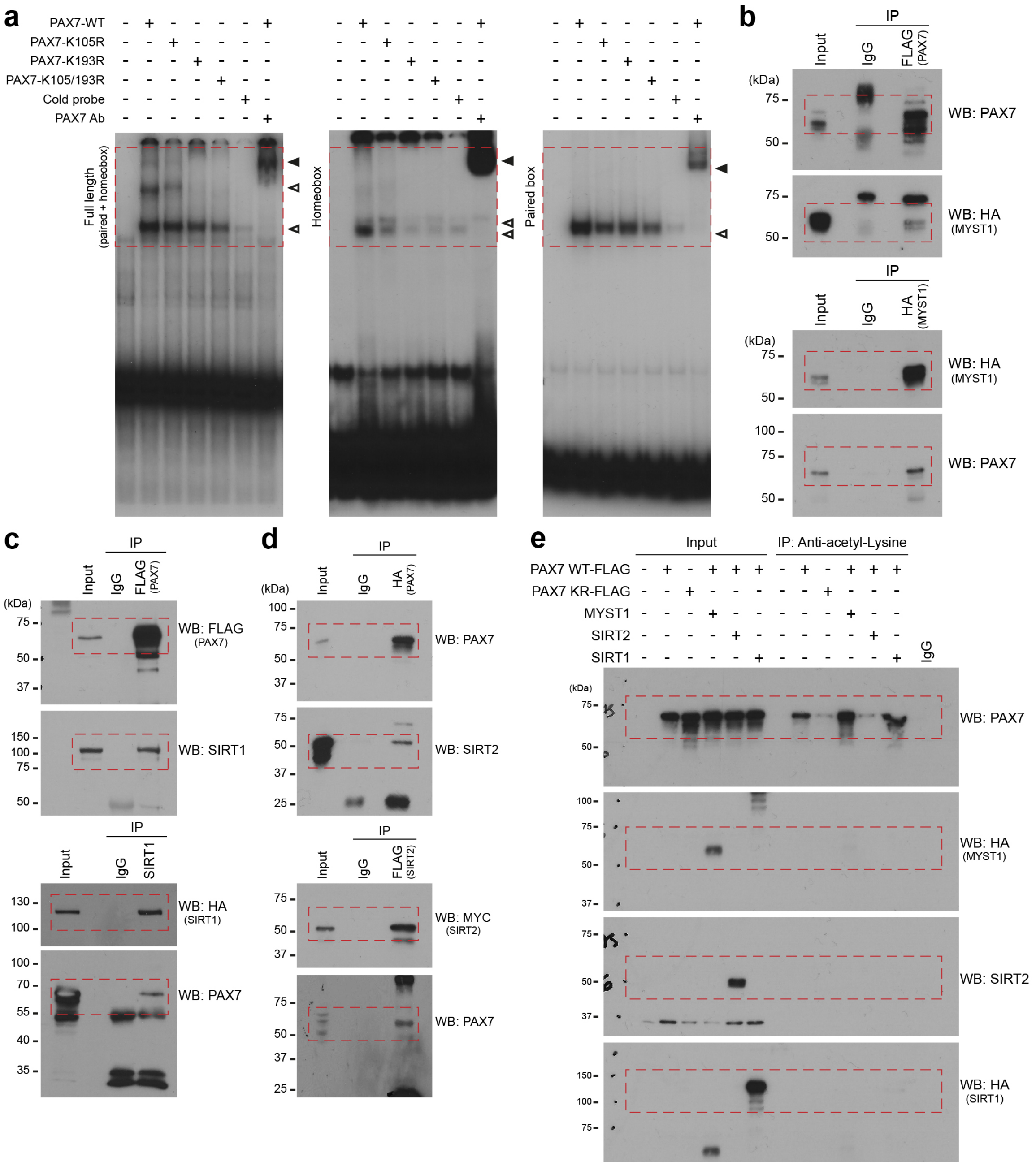

Supplementary Figure 11. Full scans of immunoblots from main Figures

a) Figure 1h

b) Figure 2a

c) Figure 2b

d) Figure 2c

e) Figure 2f

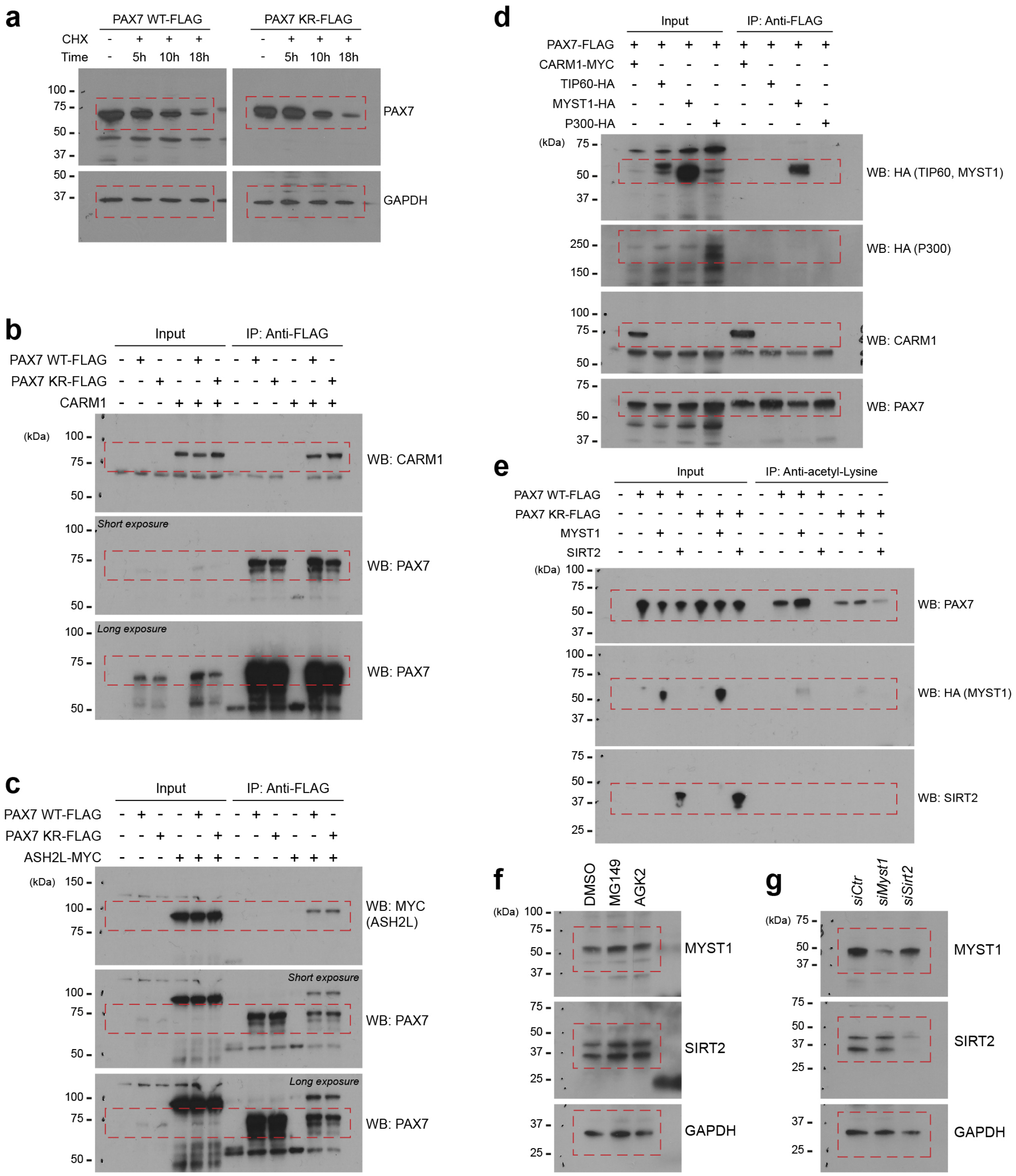

Supplementary Figure 12. Full scans of immunoblots from Supplementary Figures

a) Supplementary Figure 1a

b) Supplementary Figure 1c

c) Supplementary Figure 1d

d) Supplementary Figure 2a

e) Supplementary Figure 2b

f) Supplementary Figure 3a

g) Supplementary Figure 3b

Supplementary Table 1. List of primers used

**ChIP**	mMyf5 -111kb ChIP Fw	CATCCCACATAATCCAATCAC
	mMyf5 -111kb ChIP Rv	ACACAGATGGATGGGAAAGA
	mMyf5 -57,5kb ChIP Fw	ATACAGACATGCAGGCTTCACC
	mMyf5 -57,5kb ChIP Rv	CTCCGTATGTTTGTTGGAAAGG
**RT-qPCR**	mRps18 RT-qPCR Fw	AACGGTCTAGACAACAAGCTG
	mRps18 RT-qPCR Rv	AGTGGTCTTGGTGTGCTGAC
	mPax7 RT-qPCR Fw	GACGACGAGGAAGGAGACAA
	mPax7 RT-qPCR Rv	ACATCTGAGCCCTCATCCAG
	mMyst1 RT-qPCR Fw	GTCACACCTAAGCTGGTAGAG
	mMyst1 RT-qPCR Rv	CAGACAGAGTCCACTGTGATAG
	mSirt2 RT-qPCR Fw	GACTCCAAGAAGGCTTACA
	mSirt2 RT-qPCR Rv	CTGACTGGGCATCTATGTT
	mMyf5 RT-qPCR Fw	TGACGGCATGCCTGAATGTA
	mMyf5 RT-qPCR Rv	ATCTGCAGCACATGCATTTGATA
	mFkbp5 RT-qPCR Fw	GGCAGTCAATCCTCAGAAC
	mFkbp5 RT-qPCR Rv	CTTCTACAGCCTTCTTGCTC
	mZbtb16 RT-qPCR Fw	TAATGGCTGTGGCAAGAAG
	mZbtb16 RT-qPCR Rv	TGATCATGGCCGAGTAGT
	mUsp54 RT-qPCR Fw	CTCCTGTATTTCTCCTTGATCC
	mUsp54 RT-qPCR Rv	CATCCCTGTCACACATAGC
	mCep162 RT-qPCR Fw	GATCAAGCAGATGGAGATGAG
	mCep162 RT-qPCR Rv	TCTACTACTTGGCGTGTTTG
	mBcl6 RT-qPCR Fw	TCATCCACACTGGAGAGAA
	mBcl6 RT-qPCR Rv	GACGAAAGTGCAGGTTACA
	mPostn RT-qPCR Fw	AACCAAGGACCTGAAACACG
	mPostn RT-qPCR Rv	GTGTCAGGACACGGTCAATG
	mCamkk2 RT-qPCR Fw	GACGCTGTACTGCTTTGT
	mCamkk2 RT-qPCR Rv	GCCTGACTCTTGATCTTACTG
	mMcph1 RT-qPCR Fw	TCCAGTACCTGTCAGAGAAA
	mMcph1 RT-qPCR Rv	CATGTGACTTCAAGCCCTAA
	mMycl RT-qPCR Fw	CCTAGTCTGGAAGCCAGTAA
	mMycl RT-qPCR Rv	GTCACCACGTCAATCTCTTC
	mHdac11 RT-qPCR Fw	AGAGGAATGTCAGGAGGT
	mHdac11 RT-qPCR Rv	GTATATCATGGGCTCGGA
	mJun RT-qPCR Fw	GCAACACACAAGTCCAGA
	mJun RT-qPCR Rv	CAGCTTTCAACCAAAGTGTC
	mFos RT-qPCR Fw	GCAAAGTAGAGCAGCTATC
	mFos RT-qPCR Rv	CAACGCAGACTTCTCATC
	mMt2 RT-qPCR Fw	TCTCTCGTCGATCTTCAACC
	mMt2 RT-qPCR Rv	GGAAGCCTCTTTGCAGATG
**Generation of Pax7^{KR} mice using CRISPR-Cas9**	Pax7_gRNA_K193R	GGTTCTTACCTTTGTCGCCC
	Pax7_hdr_K193R	AGTTCGGGAAGAAAGAGGACGACGAGG AAGGAGACAAGAAAGAAGAAGATGGC GAGAAGAAAGCCAAACACAGCATCGAT GGCATtCTGGGCGACcgAGGTAAGAACCT ACCGTGGCCCGAGACGCC

**Genotyping of Pax7^{KR} mice**	Pax7 R193 MT Geno Fw	CCACGGTAGGTTCTTACCTCG
	Pax7 K193 WT Geno Rv	GGCATCCTGGGCGACAA
	Pax7 KR Geno Fw	TCGGCGATCTGCGACTTTT
	Pax7 KR Geno Rv	CCAGGAGTGAGCCAGGTTTC
**EMSA**	Myf5 -111 EMSA full Fw	GTTTTCACAATAATGCATTTTCTGTAACTAACAACCATCATTTCCTGATTGTCATGCTTCTATCCTTG
	Myf5 -111 EMSA full Rv	CAAGGATAGAAGCATGACAATCAGGAAATGATGGTTGTTAGTTACAGAAAATGCATTATTGTGAAAAC
	Myf5 -111 paired EMSA Fw	TCATTTCCTGATTGTCATGCTTCTATCCTTGCACA
	Myf5 -111 paired EMSA Rv	TGTGCAAGGATAGAAGCATGACAATCAGGAAATGA
	Myf5 -111 hbox EMSA Fw	CTGGTTTTCACAATAATGCATTTTCTGTAACTAAC
	Myf5 -111 hbox EMSA Rv	GTTAGTTACAGAAAATGCATTATTGTGAAAACCAG

Supplementary Table 2. List of antibodies used

Antibody	Company	Reference
Mouse monoclonal anti-Flag M2	Sigma-Aldrich	F3165
Rabbit polyclonal anti-c-Myc	Bethyl Laboratories	A190-105A
Rabbit anti-HA	Bethyl Laboratories	A190-108A
Rabbit anti-acetylated lysine	Abcam	ab80178
Rabbit anti-acetylated lysine	Cell Signaling Technologies	9441S
Mouse monoclonal anti-Gapdh	UBC Ablab	21-0017-16
Mouse monoclonal anti-alpha tubulin	Sigma-Aldrich	T9026
Rabbit polyclonal anti-Sirt1	EMD Millipore	07-131
Rabbit anti-Carm1	Bethyl Laboratories	A300-421A
Mouse monoclonal anti-Pax7	DSHB	N/A
Mouse monoclonal anti-Pax7	Santa Cruz Biotechnology	sc-81648
Chicken monoclonal anti-Myh1 (MF20)	DSHB	N/A
Rabbit polyclonal anti-Sirt2	St John's Labs	STJ95668
Rabbit polyclonal anti-Myst1	Bethyl Laboratories	A300-992A
Chicken anti-Syndecan-4	Brad Olwin's laboratory	
Chicken polyclonal anti-GFP	Abcam	ab13970
Rabbit anti-Dystrophin	Abcam	Ab15277
Rat monoclonal anti-Laminin-2	Sigma-Aldrich	L0663
Mouse monoclonal anti-Acetylated alpha-tubulin	Abcam	ab24610
Rat anti-alpha-7-integrin (clone R2F2) (Alexa Fluor 647-conjugated)	UBC Ablab	R2F2
Rat anti-CD31 (BV421-conjugated)	BD Biosciences	562939
Rat anti-CD45 (BV421-conjugated)	BD Biosciences	563890
Rat anti-CD11b (BV421-conjugated)	BD Biosciences	562605
Rat anti-Ly6A/E (Sca-1) (BV421-conjugated)	BD Biosciences	562729

Rat anti-CD34 (biotin-conjugated)	Miltenyi Biotech	130-105-830
Streptavidin-Pe-Cy7	BD Biosciences	557598
Rat anti-CD106 (Vcam1) (Pe-Cy7 conjugated)	Biolegend	105719

Supplementary References

1. Machado, L. *et al.* In Situ Fixation Redefines Quiescence and Early Activation of Skeletal Muscle Stem Cells. *Cell Rep* **21**, 1982-1993 (2017).
